# Aldosterone-Induced Sarco/Endoplasmic Reticulum Ca^2+^ Pump Upregulation Counterbalances Ca_v_1.2-Mediated Ca^2+^ Influx in Mesenteric Arteries

**DOI:** 10.3389/fphys.2022.834220

**Published:** 2022-03-11

**Authors:** Rogelio Salazar-Enciso, Agustín Guerrero-Hernández, Ana M. Gómez, Jean-Pierre Benitah, Angélica Rueda

**Affiliations:** ^1^Departamento de Bioquímica, Centro de Investigación y de Estudios Avanzados del IPN, Mexico City, Mexico; ^2^Signaling and Cardiovascular Pathophysiology - UMR-S 1180, Inserm, Université Paris-Saclay, Châtenay-Malabry, France

**Keywords:** calcium sparks, aldosterone (ALDO), ryanodine receptor, STOCs, Ca_v_1.2 Ca^2+^ channel, SERCA pump, mesenteric artery (MA), vascular smooth muscle cell

## Abstract

In mesenteric arteries (MAs), aldosterone (ALDO) binds to the endogenous mineralocorticoid receptor (MR) and increases the expression of the voltage-gated L-type Ca_v_1.2 channel, an essential ion channel for vascular contraction, sarcoplasmic reticulum (SR) Ca^2+^ store refilling, and Ca^2+^ spark generation. In mesenteric artery smooth muscle cells (MASMCs), Ca^2+^ influx through Ca_v_1.2 is the indirect mechanism for triggering Ca^2+^ sparks. This process is facilitated by plasma membrane-sarcoplasmic reticulum (PM-SR) nanojunctions that drive Ca^2+^ from the extracellular space into the SR via Sarco/Endoplasmic Reticulum Ca^2+^ (SERCA) pump. Ca^2+^ sparks produced by clusters of Ryanodine receptors (RyRs) at PM-SR nanodomains, decrease contractility by activating large-conductance Ca^2+^-activated K^+^ channels (BK_Ca_ channels), which generate spontaneous transient outward currents (STOCs). Altogether, Ca_v_1.2, SERCA pump, RyRs, and BK_Ca_ channels work as a functional unit at the PM-SR nanodomain, regulating intracellular Ca^2+^ and vascular function. However, the effect of the ALDO/MR signaling pathway on this functional unit has not been completely explored. Our results show that short-term exposure to ALDO (10 nM, 24 h) increased the expression of Ca_v_1.2 in rat MAs. The depolarization-induced Ca^2+^ entry increased SR Ca^2+^ load, and the frequencies of both Ca^2+^ sparks and STOCs, while [Ca^2+^]_cyt_ and vasoconstriction remained unaltered in Aldo-treated MAs. ALDO treatment significantly increased the mRNA and protein expression levels of the SERCA pump, which counterbalanced the augmented Ca_v_1.2-mediated Ca^2+^ influx at the PM-SR nanodomain, increasing SR Ca^2+^ content, Ca^2+^ spark and STOC frequencies, and opposing to hyperpolarization-induced vasoconstriction while enhancing Acetylcholine-mediated vasorelaxation. This work provides novel evidence for short-term ALDO-induced upregulation of the functional unit comprising Ca_v_1.2, SERCA2 pump, RyRs, and BK_Ca_ channels; in which the SERCA pump buffers ALDO-induced upregulation of Ca^2+^ entry at the superficial SR-PM nanodomain of MASMCs, preventing ALDO-triggered depolarization-induced vasoconstriction and enhancing vasodilation. Pathological conditions that lead to SERCA pump downregulation, for instance, chronic exposure to ALDO, might favor the development of ALDO/MR-mediated augmented vasoconstriction of mesenteric arteries.

## Introduction

Aldosterone (ALDO) is a steroid hormone that regulates the balance of water and electrolytes in the body and acts primarily through the mineralocorticoid receptor (MR), a transcription factor activated by ligand (Mangelsdorf et al., 1995). Clinical trials have evidenced the participation of MR in blood pressure (BP) regulation by showing the beneficial effects of MR blockers in the treatment of high BP; including mild, moderate and resistant hypertension (Weinberger et al., 2002; Acelajado et al., 2019). The MR is mainly expressed in kidney epithelial cells, but it is also found in several vascular tissues, including small, resistance-sized (150–300 μm in lumen diameter) mesenteric arteries (MAs) (Lombès et al., 1992; Salazar-Enciso et al., 2018), which supply blood to the gastrointestinal tract and contribute to the hypertensive process under pathological exposure to ALDO (Schiffrin, 1992). Whereas, MR, aldosterone synthase, and 11-beta hydroxysteroid dehydrogenase type 2 (11-BHSD2), key proteins of the local ALDO system, are expressed in rat MAs (Takeda et al., 1993, 1997); the impact of the ALDO/MR signaling pathway on Ca^2+^ handling proteins in mesenteric artery smooth muscle cells (MASMCs) has not been fully evaluated.

In the heart, aorta, coronary and MAs, the activation of the ALDO/MR signaling pathway increases voltage-gated L-type Ca_v_1.2 channel (LTCCs) expression (Bénitah and Vassort, 1999; Lalevée et al., 2005; Mesquita et al., 2018), resulting in enhanced vascular contraction of coronary arteries (Mesquita et al., 2018). In experimental animal models of chronic hypertension such as the spontaneously hypertensive rat (SHR), LTCCs are upregulated, Ca^2+^ influx is increased, vascular reactivity is enhanced, and receptor-stimulated contractile responses are higher than in arteries of control rats (Cox and Lozinskaya, 1995; Matsuda et al., 1997; Pratt et al., 2002; Xavier et al., 2008). However, it has been also shown that when LTCCs are activated via K^+^-mediated membrane depolarization maneuvers, vasoconstriction responses are unaffected in MAs of SHR and Wistar-Kyoto rats treated with ALDO (Xavier et al., 2008); in deoxycorticosterone (DOCA)-salt hypertensive rats (Suzuki et al., 1994); or in the young transgenic mouse with smooth muscle cell (SMC)-specific MR deficiency (SMC-MR-KO mouse) (McCurley et al., 2012); and in cerebral arteries of ALDO-treated mice (Chrissobolis et al., 2014). These studies support the notion that in some types of vascular tissues, the activation of the ALDO/MR signaling pathway enhances receptor-mediated vasoconstriction responses (for instance, α-adrenoceptor-mediated contraction) but not vasoconstriction responses induced by KCl (Suzuki et al., 1994; Xavier et al., 2008; Chrissobolis et al., 2014); and the molecular mechanisms that underlie this discrepancy are elusive.

L-type voltage-dependent Ca^2+^ channels are the primary route of Ca^2+^ entry in the vasculature (Ghosh et al., 2017). For instance, Ca^2+^ entry via LTCCs is the principal mediator of myogenic response, vascular contraction, sarcoplasmic reticulum (SR) Ca^2+^ refilling, Ca^2+^ spark generation and blood pressure in resistance-sized MAs (Nelson and Worley, 1989; Moosmang et al., 2003; Ghosh et al., 2017; Fan et al., 2018). Ca^2+^ entering the cytoplasm is captured to the SR Ca^2+^ stores by the SERCA pump at the superficial or junctional SR, which occupies a vast subcellular area with multiple plasma membrane-sarcoplasmic reticulum (PM-SR) junctions in vascular smooth muscle cells (VSMCs). In fact, LTCC-mediated subcellular Ca^2+^ signals, named Ca^2+^ sparklets, coincide with junctional SR expressing SERCA pumps and Ryanodine receptors (RyRs), demonstrating the close association of these proteins in the subplasmalemmal nanodomain (Takeda et al., 2011). In addition, the SERCA pump tightly regulates Ca^2+^ influx and as a result, indirectly controls Ca^2+^ spark ignition (Chen and van Breemen, 1993; Van Breemen et al., 1995; Essin and Gollasch, 2009). Ca^2+^ sparks, which are local Ca^2+^ signals produced by the simultaneous activation of clusters of Ca^2+^ release channels/Ryanodine receptors (RyRs), are involved in vasorelaxation (Nelson et al., 1995; Essin and Gollasch, 2009; Krishnamoorthy et al., 2014). Ca^2+^ sparks activate large-conductance Ca^2+^-activated K^+^ channels (BK_Ca_ channels) that generate spontaneous transient outward currents (STOCs) (Nelson et al., 1995; Pérez et al., 1999; Essin and Gollasch, 2009). STOCs have a key role in the control of arterial myogenic tone by shifting the plasma membrane potential toward less positive values (which limits Ca^2+^ influx through LTCCs), diminishing global cytoplasmic Ca^2+^ concentration ([Ca^2+^]_cyt_), and opposing vasoconstriction (Ganitkevich and Isenberg, 1990; Krishnamoorthy et al., 2014). Therefore, altogether Ca_v_1.2, SERCA2 pump, RyRs, and BK_Ca_ channels comprise a functional unit at the PM-SR nanodomain that regulates vascular function favoring vasorelaxation (Jaggar et al., 1998; Essin et al., 2007; Essin and Gollasch, 2009; Van Breemen et al., 2013).

Accessory proteins of RyRs (for instance, FKBP12.6, sorcin, and calsequestrin-2) (Wang et al., 2004; Rueda et al., 2006; Esfandiarei et al., 2013), might also participate in regulating this functional unit, but whether ALDO treatment alters their expression and activity in MAs is unknown.

In this work, we determined the effect of a short-term (24 h) exposure to aldosterone (ALDO) in the expression and activity of Ca_v_1.2, SERCA2 pump, RyR, and BK_Ca_ channels, proteins that regulate intracellular Ca^2+^ handling and vascular function of mesenteric arteries.

## Materials and Methods

All procedures were performed according to the ethical guidelines of the Mexican Official Norm (NOM-062-ZOO-1999) and the National Institutes of Health Guide for the Care and Use of Laboratory Animals (NIH publication updated in 2011). The animal protocol was approved by the Institutional Bioethical Committee for Care and Handling of Laboratory Animals at the Cinvestav-IPN (approved CICUAL Protocol No. 0100-14). Unless specified, all reagents were purchased from Sigma–Aldrich Quimica, S. de RL. de C.V., Toluca, Mexico.

### Dissection of Mesenteric Arteries and Aldosterone Treatment

Twelve-week-old male Wistar rats (250–300 g of body weight) were anesthetized by intraperitoneal injection of sodium pentobarbital solution (100 mg/Kg of body weight. Pisabental^®^ PISA agropecuaria S.A. de C.V. Tula, Hidalgo, Mexico). To rule out the effects of chronic ALDO-mediated maladaptive vessel changes, and to avoid unsought Angiotensin II-induced vascular MR activation (Jaffe and Mendelsohn, 2005); MAs were isolated and *ex vivo* exposed to 10 nM ALDO for 24 h, as previously described (Mesquita et al., 2018). Briefly, third-order, resistance-sized MAs were dissected under the microscope in ice-cold HEPES-buffered dissection solution (in mM: 80 Na-Glutamate, 55 NaCl, 6 KCl, 2 MgCl_2_, 10 glucose, 10 HEPES; pH 7.4 with NaOH). Arteries were cleaned of fat and connective tissue and transferred to free-serum Dulbecco’s Modified Eagle Medium (DMEM, Gibco™ Cat# 11885084, Thermo Fisher Scientific Inc., Waltham, MA, United States) supplemented with penicillin and streptomycin as previously described (Mesquita et al., 2018). Artery segments were cultured in a humidified atmosphere of 5% CO_2_ at 37°C for 24 h in the presence of 10 nM aldosterone (ALDO group) or in its absence (control group). After incubation, MAs were used for vascular reactivity experiments or stored at −80°C for Western Blots and real-time qPCR experiments.

### Vascular Reactivity Assays

Vascular reactivity was assessed in third-order, resistance-sized MAs using a wire myograph for small vessels (Danish Myotechnology, Aarhus, Denmark) as previously described (Mesquita et al., 2018) with some modifications. After treatment, MAs were cut into 1.5–2.0 mm rings, cannulated with two 40 μm diameter stainless steel wires and mounted in an organ bath, warmed at 37°C with physiological saline solution (PSS) containing (in mM): 119 NaCl, 4.7 KCl, 2.5 CaCl_2_, 1.17 MgSO_4_, 1.18 KH_2_PO_4_, 25 NaHCO_3_, 11 glucose and continuously gassed with carbogen (95% O_2_, 5% CO_2_) to maintain pH at 7.4. The MA rings were stabilized at a tension equivalent to that generated at 0.9× the diameter of the vessel at 100 mmHg for 45 min before experimentation. To determine the ring viability and achieve the maximal contractile response, MA rings were challenged with 60 mM KCl-containing PSS solution (equimolar substitution with NaCl to maintain constant ionic strength) twice before the beginning of concentration-response curves (KCl, from 10 to 60 mM). Endothelium integrity was tested using an endothelium-dependent agonist, Acetylcholine (ACh). The vasorelaxation response was determined by concentration-response curves (ACh, from 10^–9^ to 10^–5^ M) in pre-contracted MA rings with 60 mM KCl. Each vasoconstriction or vasorelaxation experiment was performed in duplicate, with the mean used as a single experimental value. Data are shown as the percentage of maximal contractile response elicited by 60 mM KCl-containing solution, which was considered 100%.

### Preparation of Mesenteric Artery Homogenates, Sodium Dodecyl Sulfate-Polyacrylamyde Gel Electrophoresis and Immunoblot Analyses

Protein expression levels were assessed by immunoblotting as previously reported (Rueda et al., 2013; Mesquita et al., 2018) with some modifications. Briefly, 3–4 MA segments pooled from three rats were pulverized in liquid N_2_ and homogenized on ice with a glass tissue grinder (Potter-Elvehjem) containing 200 μl cold homogenization buffer (composition in mM: 20 HEPES, 20 NaF, 300 sucrose, plus 0.5% sodium deoxycholate, and 0.1% SDS, pH 7.2 with NaOH) supplemented with protease inhibitors (1 μg/ml aprotinin, 500 μM benzamidine, 12 μM leupeptin, 100 μM PMSF). Homogenates were centrifuged at 2,000 × *g* for 10 min at 4°C and the supernatant was collected. Protein concentration was determined by the Bradford method. Supernatants were fractionated on 4–12% discontinuous gradient SDS-PAGE gel (20 μg of protein per well, for 2 h at 90 V). Separated proteins were transferred onto nitrocellulose or PVDF membrane for 2 h, 100 V at 4°C and blocked from non-specific binding with 5% non-fat dried milk in phosphate buffered saline-Tween 20 (0.1%) (PBS-T) for 1 h, before the incubation with commercial primary antibodies previously used at indicated publications, against Ca_v_1.2 (1:200, Cat# AB10515, Millipore, Merck KGaA, Darmstadt, Germany) (Mesquita et al., 2018); SERCA2 pump (1:4,000, Cat# ab2861, Abcam, Cambridge, MA, United States) (Romero-García et al., 2020); Ryanodine receptor (RyR, 1:5000, Cat# ab2868, Abcam, Cambridge, MA, United States) (Rueda et al., 2006); calsequestrin (CSQ2, 1:4,000, Cat# ab108289, Abcam, Cambridge, MA, United States) (de Alba-Aguayo et al., 2017); sorcin (1:1,000, a kind gift from Héctor H. Valdivia laboratory, University of Wisconsin, Madison, WI, United States) (Rueda et al., 2006); FKBP12.6 (1:2,000, Cat# sc-376135, Santa Cruz Biotechnology, Inc., Dallas, TX, United States) (Gómez et al., 2009); MR (1:200; Cat# MRN2 2B7, DSHB, University of Iowa, Iowa City, IA, United States) (Gomez-Sanchez et al., 2006); BK_Ca_ α subunit (1:200, Cat# APC-009, Alomone Labs, Jerusalem, Israel) (Rueda et al., 2013), BK_Ca_ β1 subunit (1:5000, Cat# APC-036, Alomone Labs, Jerusalem, Israel) (Rueda et al., 2013), Orai1 (1:200, Cat# O8264, Sigma–Aldrich Química, S.L. Toluca, Mexico) (Bartoli et al., 2020) and GAPDH (1:100,000 Cat# AM4300, Ambion^®^, Thermo Fisher Scientific Inc., Waltham, MA, United States) (Romero-García et al., 2020) for 2 h at room temperature (RT). Membranes were incubated for 1 h at 37°C with horseradish peroxidase-conjugated anti-rabbit y/or anti-mouse in PBS-T with 1% non-fat dried milk. Signal was detected by chemiluminescence on radiograph film. The density of immunoreactive bands was measured on KODAK Image Station. GAPDH was used as loading control.

### Total RNA Isolation and Real-Time Quantitative Polymerase Chain Reaction

Total RNA was extracted from pools of MAs from 3 to 4 animals by TRIzol reagent^®^ according to the manufacturer’s protocol. Total RNA was digested for 20 min at 37°C with 0.5 μL DNase (Roche, Mannheim, Germany). The reverse transcription was performed with Superscript II Reverse Transcriptase KIT (Invitrogen). The resulting DNA samples were amplified by real-time qPCR using the QuantiTect SYBR ^®^Green PCR Kits (QIAGEN) in a Rotor-Gene cycler (Corbett Research, Sydney, NSW, Australia). mRNA levels were normalized to the geometric mean of three housekeeping genes, 60S ribosomal protein L32 (*Rpl32*), 14-3-3 protein zeta/delta (*Ywhaz*), and transgelin (*Sm22*) (Mesquita et al., 2018). All samples were quantified in triplicate. The values were expressed as a relative expression using the Pfaffl equation (Pfaffl, 2001). The specific primers sequences are listed in Table 1.

**TABLE 1:** List of primer sequences for real-time qPCR.

Target gene	Sequence accession number	Primer	Tm (°C)	Sequence	Product length (pb)
*Atp2a2*	NM_001110139.2	F R	59.0 59.1	5′- ACCTGGAAGATTCTGCGAAC -3′ 5′- AATCCTGGGAGGGTCCAG -3′	86
*Sgk1*	NM_019232.3	F R	60.3 58.4	5′- CTGCTCGAAGTACCCTCACC -3′ 5′- GCATGCATAGGAGTTGTTGG -3′	128
*Orai1* *Stim1*	NM_001013982.1 NM_001108496.2	F R F R	59.49 59.88 59.54 59.63	5′- ATCGTCTTTGCCGTTCACTT -3′ 5′- AGAGAATGGTCCCCTCTGTG -3′ 5′- TCTCTGAGTTGGAGGATGAGTAGA -3′ 5′- CAATATAGGGGAGCAGAGGTAAGA -3′	131 112
*Tagln*	NM_031549.2	F R	60.5 62.2	5′- GTTTGGCCGTGACCAAGAAC -3′ 5′- GGAGGCCAATGACGTGCTTC -3′	129
*Rpl32*	NM_013226.2	F R	58.6 58.9	5′- GCTGCTGATGTGCAACAAA -3′ 5′- GGGATTGGTGACTCTGATGG -3′	115
*Ywhaz*	NM_013011.3	F R	60.3 54.7	5′- AGACGGAAGGTGCTGAGAAA -3′ 5′- GAAGCATTGGGGATCAAGA -3′	127

*Atp2a2, ATPase sarcoplasmic/endoplasmic reticulum Ca^2+^ transporting 2 or SERCA pump; Sgk1, serum/glucocorticoid-regulated kinase; Orai1, calcium release-activated calcium channel protein 1; Stim1, stromal interaction molecule 1; Tagln, transgelin also known as smooth muscle cell 22 alpha; Rpl32, ribosomal protein L32; Ywaz, tyrosine 3-monooxygenase/tryptophan 5-monooxygenase activation protein, zeta; F, forward; R, reverse; Tm, melting temperature; pb, pair bases. Information obtained from MFEprimer 3.1 (Wang et al., 2019).*

### Mesenteric Artery Smooth Muscle Cell Isolation

Single smooth muscle cells from mesenteric arteries (MASMCs) were enzymatically isolated following as previously described (Rueda et al., 2013) with some modifications. Briefly, MAs were cut into small segments and transferred to a 1-ml aliquot of PSS containing (in mg/ml): 1 papain, 1 bovine serum albumin (BSA), 1 dithiothreitol for 16 min at 37°C. The digested tissue was transferred to a 1 ml PSS supplemented with collagenase type F (1 mg/ml) plus 100 μM CaCl_2_ for 8 min at 37°C. The digestion was stopped by three washes with cold PSS. Individual cells were dissociated from vessels by gentle trituration with a fire polish glass pipette. The resulting cell suspension was stored at 4°C for up 1 h and was used on the same day. Only long, smooth, and optically refractive cells were used.

### Cytoplasmic Ca^2+^ Concentration Measurements in Single Mesenteric Artery Smooth Muscle Cells

The global cytoplasmic Ca^2+^ concentration ([Ca^2+^]_cyt_) and the amplitude of caffeine-induced Ca^2+^ transients were estimated as previously described (Rueda et al., 2002) with some modifications. Briefly, isolated MASMCs were loaded with 8 μM Fura 2-AM in 20 mM K^+^-physiological saline solution (PSS-20K, composition in mM: 122 NaCl, 20 KCl, 2 CaCl_2_, 1 MgCl_2_, 10 glucose, 10 HEPES, pH 7.4 with NaOH) for 25 min at RT and washed. An aliquot of 20 μL of Fura 2-loaded cells was placed in a 1-mL-volume recording chamber (homemade) filled with PSS-20K at RT. Cells were allowed to adhere to the chamber’s glass bottom for at least 5 min. Fura-2 fluorescence excitation ratios (*F*_340_/*F*_380_) were determined in individual cells each 50 ms within a 100-μm^2^ recording window in an inverted microscope (Nikon Diaphot, Japan) coupled to a PTI microfluorometry system (Ratio-Master™ PTI Technologies Inc., South Brunswick, NJ, United States). A single and brief pulse (10 s) of 10 mM caffeine (Caff) was locally applied by a pneumatic pump (PV830 PicoPump, WPI, Sarasota, FL, United States) using a TW100F-4 borosilicate micropipette placed above the cell. Data acquisition and analyses were performed in FeliX32 software (PTI Technologies Inc., South Brunswick, NJ, United States). The Fura-2 fluorescence ratio values (*F*_340_/*F*_380_) were converted to [Ca^2+^] with the Grynkiewicz equation: *[Ca^2+^]* = K_d_*β *(R-R_*min*_/R_*max*_-R)* (Grynkiewicz et al., 1985); where *R* is the ratio (*F*_340_/*F*_380_) after background subtraction. Values of 0.21, 6.25, and 9.7 obtained from Fura-2 *in situ* calibration were used for *R*_*min*_, *R*_*max*_, and β, respectively; *R*_*min*_ and *R*_*max*_ were obtained in the presence of 2 mM EGTA and 2.5 mM extracellular Ca^2+^, respectively. *K*_*d*_ used was 282 nM.

### Determination of Ca^2+^ Influx

Fluo 4-loaded MASMCs were kept in a Na^+^ and Ca^2+^ free solution (in mM: 136 LiCl, 6 KCl, 2 MgCl_2_, 10 glucose, 10 HEPES, 1 EGTA; pH 7.4 with LiOH), and incubated with either thapsigargin (TGN, 100 nM) or Nifedipine (1 μM) for 10 min at RT. Subsequently, cells were perfused with a Na^+^ free solution containing 1.8 mM Ca^2+^. Changes in cytoplasmic Ca^2+^ were recorded with a laser scanning confocal microscope (Zeiss, LSM 700) equipped with an ×63 oil immersion objective (N.A. 1.2). Fluo-4 was excited at 488 nm with a diode laser of 488 nm (5% intensity), and emitted light was directed onto a main dichroic beam splitter (MDBS) to separate the emission from the excitation light. The emitted light was diverted to a secondary dichroic (VSD) beam splitter which in combination with a long pass filter (LP515) allowed the collection of light by the photomultiplier (PMT) above 510 nm. After subtracting background fluorescence (calculated outside of the cell), Fluo 4 fluorescence signals were normalized by dividing the fluorescence intensity of each pixel (*F*) by the average basal fluorescence intensity inside the cell (*F*_0_). Fluo 4 fluorescence signals corresponding to the response after the reintroduction of external Ca^2+^ were reported as Δ*F*/*F*_0_.

### Ca^2+^ Sparks Recordings

The local Ca^2+^ release events were recorded as previously described (Rueda et al., 2013) with some modifications. Intact MA segments were loaded with the Ca^2+^ indicator Fluo 4-AM (10 μM final concentration, 40 min) prepared in PSS-20K. The MA segments were allowed to adhere to the bottom of a glass coverslip in a perfusion chamber and were perfused with PSS-20K at RT. Ca^2+^ sparks were recorded with a laser scanning confocal microscope (Zeiss, LSM 700, Carl Zeiss de México, S.A. de C.V.) equipped with an ×63 oil immersion objective (N.A. 1.2) in *line scan* mode (five images per cell of 1000 lines each, at speed of 1.92 ms/line). Fluo-4 was excited at 488 nm with a diode laser (3% intensity) and emitted light was directed onto a MDBS to separate the emission from the excitation light. The emitted light was diverted to a VSD beam splitter which in combination with a LP515 allowed the collection of light by the PMT above 510 nm. Images of Ca^2+^ sparks were normalized by dividing the fluorescence intensity of each pixel (*F*) by the average basal fluorescence intensity (*F*_0_) inside the cell to generate an *F*/*F*_0_ image. Ca^2+^ spark frequency was reported as the number of events recorded per cell in five *line-scan* images. Ca^2+^ spark properties of amplitude (*F*/*F*_0_), duration (FDHM, full duration at half maximum, in ms), and width (FWHM, full width at half maximum, in μm) were measured with a custom-made program running in IDL 5.5 software (Research Systems Inc.) (Rueda et al., 2013).

### Spontaneous Transient Outward Currents Recordings

Spontaneous transient outward currents (STOCs) were recorded with the patch-clamp technique in whole-cell configuration, as previously reported (Rueda et al., 2013). Polish fire patch pipettes (3–5 MΩ) were filled with internal solution containing (in mM): 80 K-glutamate, 5 NaCl, 40 KCl, 2 MgCl_2_, 2 Mg_2_-ATP, 0.1 Na-GTP, 0.05 K-EGTA, 20 HEPES, pH 7.2 with KOH. MASMCs were perfused with PSS and voltage-clamped from −60 to 0 mV to record STOCs. Membrane capacitance (Cm) was determined from the current amplitude elicited in response to a hyperpolarizing pulse from a holding potential at −60 mV (duration, 10 ms; amplitude, 10 mV). Currents were filtered at 500 Hz and digitalized at 2 kHz (500 μs/point). STOCs analysis was performed offline, using the event detection tool of Clampfit 9.2 (Axon Instruments, Inc.). All experiments were done at RT. Only smooth, elongated, spindle shape and optically refractive cells were used for patch experiments.

### Statistical Data Analysis

Data are presented as the mean ± standard error of the mean (M ± SEM). The number of animals for each experiment was indicated by “*N*,” while the number of vessels, cells, or independent experiments was indicated as “*n*.” All analyses were performed using Origin Pro v.8 software (Origin Lab Corporation, Northampton, MA, United States) or Sigma Plot v11.0 (Systat Software Inc., San Jose, CA, United States). After checking for normal distribution of data with the Shapiro–Wilk test, statistical significance was evaluated by either Student’s *t*-test or one-way RM Analysis of Variance (ANOVA) followed by Bonferroni *post-hoc* test. When data sets failed the normality distribution test, statistical significance was evaluated by either Mann–Whitney rank sum test or Kruskal–Wallis one-way ANOVA on ranks followed by Bonferroni *post-hoc* test. A *P*-value of ≤0.05 was considered statistically significant.

## Results

### Increased Expression of Ca_v_1.2 Does Not Enhance Depolarization-Induced Vasoconstriction in Aldosterone-Treated Mesenteric Arteries

**FIGURE 1:**
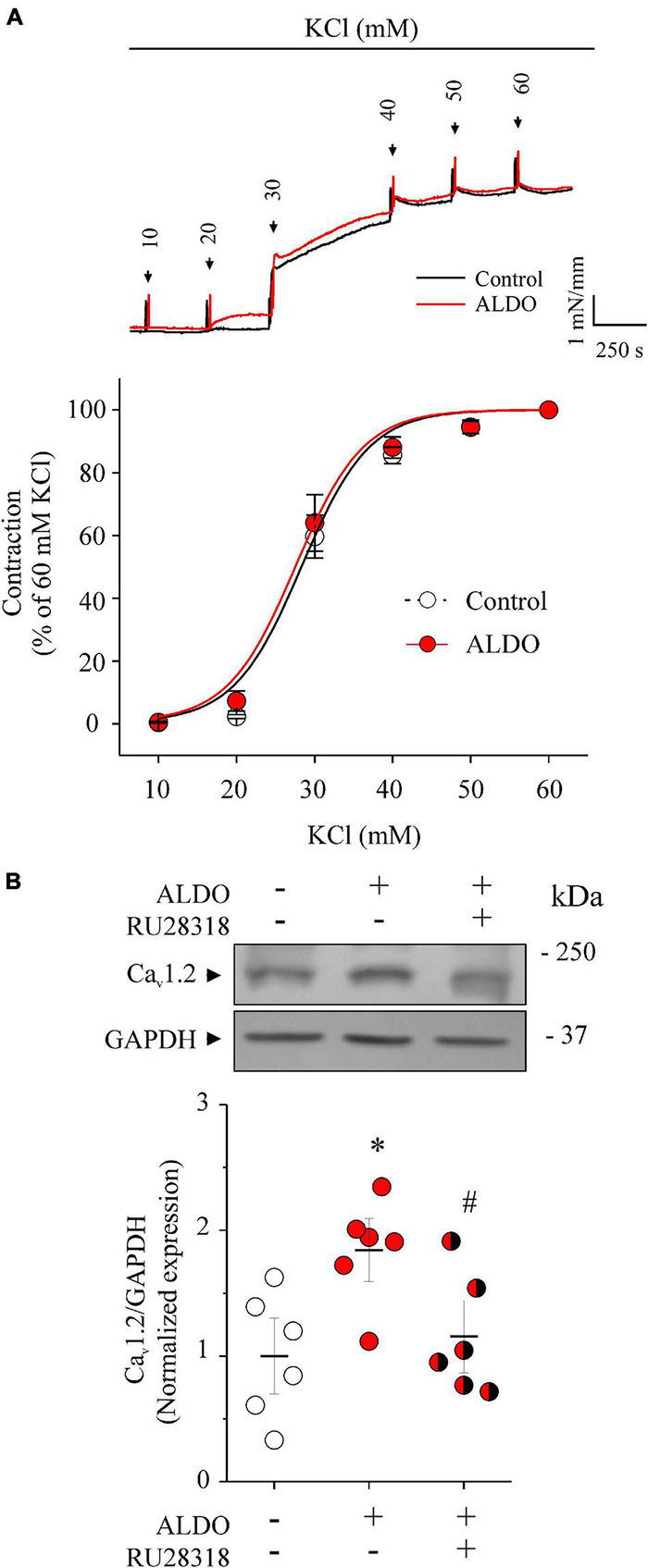
Aldosterone treatment increases Ca_v_1.2 expression but does not change depolarization-induced vasoconstriction responses of rat mesenteric arteries. **(A)** Representative traces of tension changes (*top recordings*) in response to potassium chloride (KCl) additions (from 10 to 60 mM KCl) of rat MA rings treated with aldosterone (ALDO 10 nM, 24 h, *red trace*) or without it (Control, *black trace*). The concentration-response graph (*below*) shows vasoconstriction responses of MA rings treated (*red circles*) or not (*empty circles*) with ALDO (10 nM, 24 h). Depolarization-induced contraction was expressed as a percentage of maximal contractile response induced by 60 mM KCl. Data are shown as mean ± SEM of *n* = 7 MA rings from *N* = 7 rats for each experimental group. **(B)** Representative immunoblot image and scatterplot with mean ± SEM (*below*) of Ca_v_1.2 protein expression from homogenates of control MAs (*empty circles*), ALDO-treated MAs (*red circles*), and ALDO-treated MAs co-incubated with RU 28318, a selective MR antagonist (1 μM, *red/black circles*). Data are shown as mean ± SEM of *n* = 6 independent experiments for each group. Each experiment was performed with a pool of 3–4 MA segments from three rats. *P < 0.01 vs. untreated MAs. ^#^*P* < 0.05 vs. ALDO-treated MAs.

We have reported previously that ALDO-induced MR activation increased Ca_v_1.2 expression in rat vascular tissues, including resistance MAs (Mesquita et al., 2018). However, the functional outcome of the ALDO-induced Ca_v_1.2 increased expression remained uncharacterized in MAs. Herein, we first evaluate the effect of depolarization-induced vasoconstriction in MAs isolated from male *Wistar* rats treated or not with ALDO (10 nM, 24 h). Figure 1A shows representative KCl-induced contractile responses of MA rings treated or untreated with ALDO. The vasoconstriction response of ALDO-treated MA was similar to those of control MAs (EC_50_ for vasoconstriction in mM: 28.87 ± 0.62, *n* = 7 control arteries vs. 29.64 ± 0.45, *n* = 7 ALDO-treated arteries; *P* = 0.9534). This result contrasts with previous data, that ALDO increased the depolarization-induced vasoconstriction response of coronary arteries (Mesquita et al., 2018). This observation prompted us to measure the endogenous expression of MR and its functional activity in resistance MAs. As reported previously (Takeda et al., 1993, 1997), we corroborate the constitutive expression of MR in rat MAs (Supplementary Figure 1). Furthermore, ALDO treatment induced the expression of the serum and glucocorticoid-induced kinase 1 (*Sgk1*), an enzyme that increases its expression in response to ALDO-MR activation in MAs (Briet et al., 2016). Our results demonstrate that the endogenous MR is functional in *ex vivo*-treated MAs.

In addition, we confirmed the specificity of MR activation for inducing the expression of Ca_v_1.2 in MAs treated with ALDO. Figure 1B shows representative immunoblots of Ca_v_1.2 and GAPDH (as the loading control) from ALDO-treated (10 nM, 24 h) and untreated MAs. Dispersion data graph (*below*) showed a 1.8-fold increase in Ca_v_1.2 expression in MAs incubated with ALDO. Importantly, the addition of 1 μM RU28318, a selective MR antagonist (Kim et al., 1998), blocked the effect of ALDO on Ca_v_1.2 expression.

### Aldosterone Increases Sarcoplasmic Reticulum Ca^2+^ Content but Does Not Elevate Global Cytoplasmic Ca^2+^ Concentration in Mesenteric Artery Smooth Muscle Cells

**FIGURE 2:**
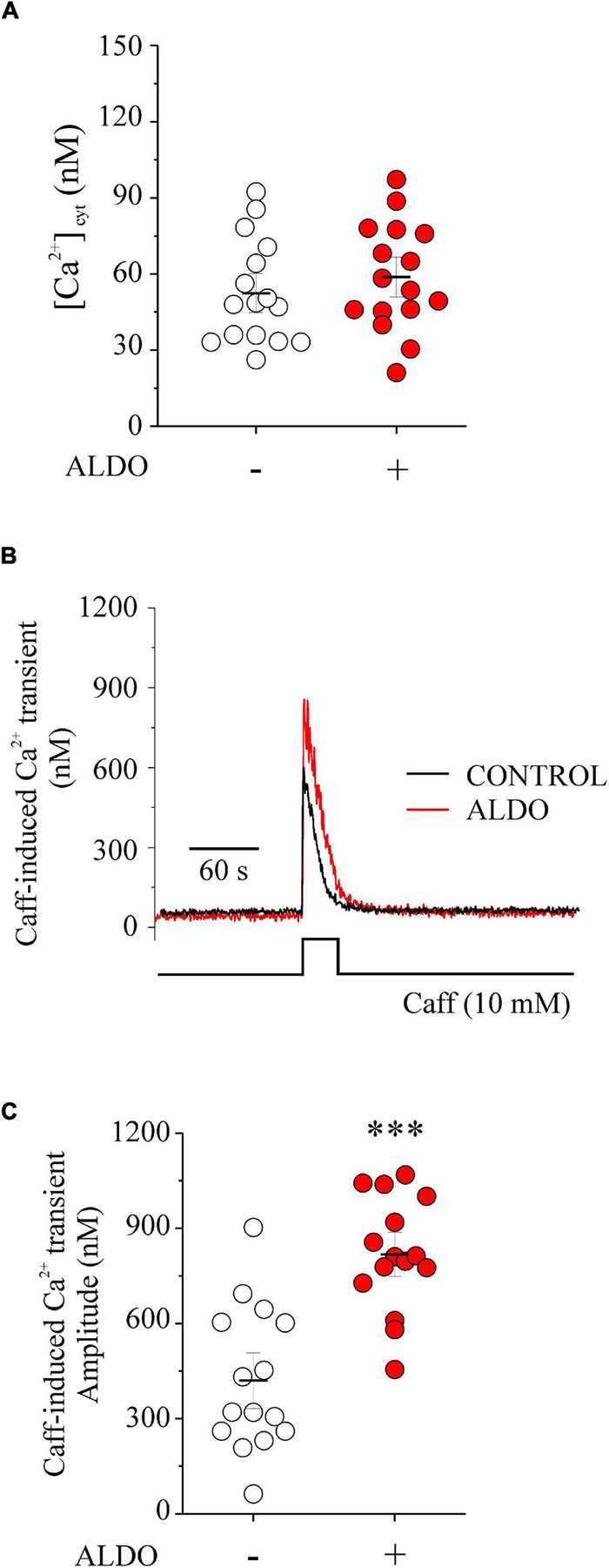
Aldosterone treatment increases SR Ca^2+^ content and does not change global [Ca^2+^]_cyt_ in Fura 2-loaded MASMCs. **(A)** Scatterplot with mean ± SEM illustrates basal [Ca^2+^]_cyt_ before caffeine challenge determined in Fura 2-loaded MASMCs, treated (*red circles*, *n* = 16 cells/*N* = 5 rats) or not (*empty circles*, *n* = 16 cells/*N* = 5 rats) with aldosterone (ALDO 10 nM, 24 h). **(B)** Representative recordings of caffeine-induced [Ca^2+^] transients in Fura 2-loaded MASMCs treated (ALDO, *red trace*) or not (CONTROL, *black trace*) with aldosterone (10 nM, 24 h). **(C)** Scatterplot with mean ± SEM of caffeine-induced Ca^2+^ transient amplitude recorded at the peak of the Ca^2+^ transient in MASMCs treated (*red circles*, *n* = 16 cells/*N* = 5 rats) or not (*empty circles*, *n* = 16 cells/*N* = 5 rats) with ALDO. ^***^P < 0.001 vs. control MASMCs.

Previous evidence suggests a dual role of Ca_v_1.2-mediated Ca^2+^ entry in VSMCs by contributing to increase global [Ca^2+^]_cyt_ and vasoconstriction (Moosmang et al., 2003), and to refill SR Ca^2+^ stores, supporting Ca^2+^ sparks and vascular relaxation (Essin et al., 2007; Fan et al., 2018). The lack of ALDO effect in enhancing depolarization-induced vasoconstriction of MAs, despite inducing an augmented Ca_v_1.2 expression (Figure 1), prompted us to hypothesize that Ca_v_1.2 might provide Ca^2+^ primarily for loading SR Ca^2+^ stores. To determine whether the increase in Ca_v_1.2 expression had an impact on the intracellular Ca^2+^ levels, we evaluated the effect of ALDO on global [Ca^2+^]_cyt_ and SR Ca^2+^ content. The latter with a challenge of caffeine (Caff, 10 mM) in Fura 2-loaded MASMCs. Figure 2A shows that global [Ca^2+^]_cyt_ was similar in ALDO-treated and untreated, controls cells, even under mild depolarizing conditions (bath solution containing 20 mM K^+^). Therefore, Ca^2+^ influx due to depolarization-induced Ca_v_1.2 activation did not increase global [Ca^2+^]_cyt_ significantly. Then, we evaluated the SR Ca^2+^ content by a single caffeine challenge. Figure 2B shows representative Caff-evoked Ca^2+^ transients in MASMCs treated or not with ALDO. Summarized data show a significantly higher amplitude of the caffeine-induced Ca^2+^ transient in MASMCs treated with ALDO with respect to control cells (Figure 2C). In addition, the area-under-the-curve (AUC) of the Caff-induced Ca^2+^ transient was significantly larger in the ALDO-treated MASMCs (AUC in arbitrary units: 7,564 ± 775 in ALDO-treated group vs. 5,448 ± 630 in CONTROL group; *P* ≤ 0.05; *n* = 15 cells for each experimental condition). These results suggest that mild depolarizing conditions activate Ca_v_1.2-mediated Ca^2+^ influx, which mainly contributes to increasing SR Ca^2+^ stores instead of increasing global [Ca^2+^]_cyt_. In the presence of the SERCA pump inhibitor thapsigargin (TGN, 100 nM) no increase in [Ca^2+^]_cyt_ was produced by an additional caffeine challenge (Supplementary Figure 2). Therefore, when SR Ca^2+^ stores are empty, the application of solution with the puffer pipette is neither releasing more Ca^2+^ from other Ca^2+^ storage organelles nor activating transient receptor potential (TRP) channels involved in mechanosensitive-mediated Ca^2+^ entry (for instance, TRPC6 and TRPP2) (Ghosh et al., 2017).

### Aldosterone Treatment Increases Ca_v_1.2-Mediated Ca^2+^ Entry but the Sarco/Endoplasmic Reticulum Ca^2+^ Pump Impedes Ca^2+^ Reaching the Bulk of the Cytoplasm of Mesenteric Artery Smooth Muscle Cells

**FIGURE 3:**
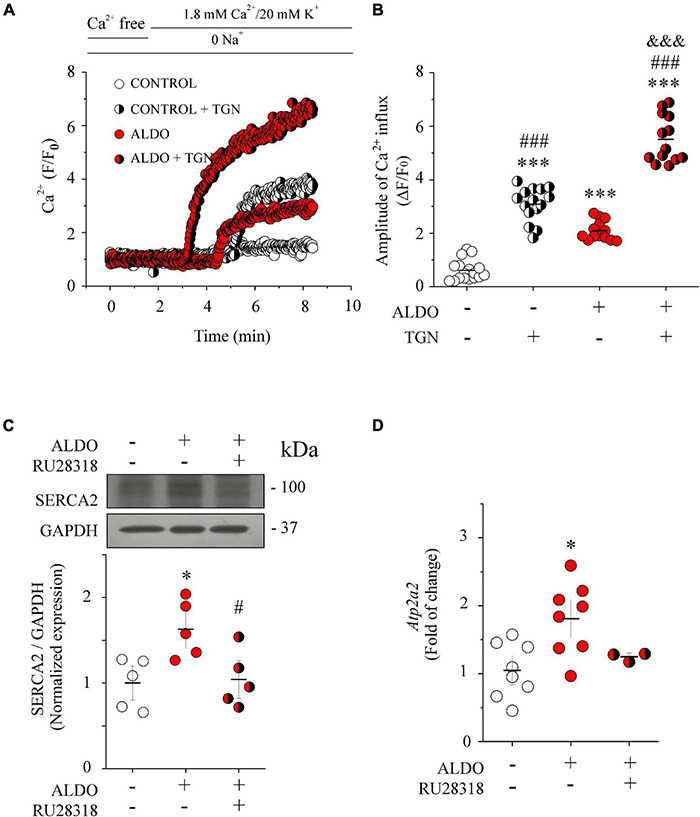
SERCA pump counterbalances depolarization-induced Ca^2+^ entry in ALDO-treated MASMCs. **(A)** Representative Ca^2+^ influx recordings (*F*/*F*_0_) of Fluo 4-loaded MASMCs from control (*empty circles*) or ALDO-treated (*red circles*) cells preincubated with thapsigargin (TGN) 100 nM for 10 min (*white/black* and *red/black circles*, respectively) to block SERCA pump activity, or in its absence. Cells were kept in Na^+^ and Ca^2+^ free solution. After 2-min of *frame-scan* recording, cells were perfused with Na^+^ free solution (to block Na^+^/Ca^2+^ exchanger activity) containing 1.8 mM CaCl_2_ plus 20 mM KCl to induce LTCC-mediated Ca^2+^ influx. Changes in cytoplasmic Ca^2+^ were recorded with a laser scanning confocal microscope (Zeiss, LSM 700) equipped with an ×63 oil immersion objective (N.A. 1.2). **(B)** Scatterplot with mean ± SEM illustrates the amplitude of depolarization-induced Ca^2+^ entry (Δ*F*/*F*_0_) of Fluo 4-loaded MASMCs under the different experimental conditions. The amplitude of Ca^2+^ influx was determined at minute 8 of the recording in control MASMCs (*n* = 17 cells/*N* = 4 rats, *empty circles*), ALDO-treated MASMCs (*n* = 13 cells/*N* = 5 rats, *empty circles*), control MASMCs + TGN (*n* = 15 cells/*N* = 6 rats, *white/black circles*) and ALDO-treated MASMCs + TGN (*n* = 13 cells/*N* = 5 rats, *red/black circles*), respectively. ^***^P < 0.001 vs. control cells; ^###^*P* < 0.001 vs. ALDO-treated cells; ^&&&^*P* < 0.001 vs. control + TGN cells. **(C)** Representative immunoblot image and scatterplot with mean ± SEM (*below*) of SERCA pump protein expression from homogenates of control MAs (*empty circles*), ALDO-treated MAs (*red circles*), and ALDO-treated MAs co-incubated with 1 μM RU28318, a selective MR antagonist (*red/black circles*). Data are shown as mean ± SEM of *n* = 5 experiments for each group. Each experiment was performed with a pool of 3–4 MA segments from three rats. SERCA pump expression levels were normalized to the expression of GAPDH for each independent experiment. *P < 0.05 vs. control group. ^#^*P* < 0.05 vs. ALDO-treated group. **(D)** Scatterplot with mean ± SEM of *Atp2a2* relative mRNA levels determined by real-time qPCR from control MAs (*empty circles*, *n* = 8), ALDO-treated MAs (*red circles*, *n* = 8), and ALDO-treated MAs co-incubated with 1 μM RU28318 (*red/black circles, n* = 3). *P < 0.05 vs. control group.

Sarco/Endoplasmic reticulum Ca^2+^ pump transports Ca^2+^ ions from the cytoplasm into the SR and contributes to maintaining luminal SR Ca^2+^ levels (Flores-Soto et al., 2013). The augmented SR Ca^2+^ load in ALDO-treated cells suggests that SERCA pump was involved in buffering Ca^2+^ coming from the extracellular space, preventing the increase in global [Ca^2+^]_cyt_. To unmask the effect of the SERCA pump, we measured Ca^2+^ entry in MASMCs preincubated with thapsigargin (TGN 100 nM, 10 min) or in its absence. Figure 3A shows representative recordings of Ca^2+^ entry in Fluo 4-loaded MASMCs under all experimental conditions. After maintaining cells in the Na^+^ and Ca^2+^ free solution, the reintroduction of extracellular Ca^2+^ increased cytoplasmic Ca^2+^ in both CTRL and ALDO-treated cells with respect to basal Ca^2+^ levels. Importantly, the increase in cytoplasmic Ca^2+^ level was larger in ALDO-treated cells preincubated with TGN (5 min) than in cells without the SERCA inhibitor (Δ*F*/*F*_0_: 0.6 ± 0.1, *n* = 15 control cells, vs. 3.1 ± 0.2, *n* = 17 control + TGN cells; and 2.1 ± 0.1, *n* = 13 ALDO-treated MASMCs vs. 5.5 ± 0.1, *n* = 13 ALDO-treated + TGN cells. Figure 3B), unmasking SERCA buffering activity. We measured the protein and mRNA levels of the SERCA pump by Western blot and qPCR, respectively (Figures 3C,D). Both were significantly increased in the ALDO group (1.7 and 1.8 folds, respectively), and blunted in the presence of RU28318 (Figures 3C,D). Given that TGN diminishes luminal SR Ca^2+^ load and stimulates store-operated Ca^2+^ entry (SOCE) (Leung et al., 2008; Trebak and Putney, 2017); and also that ALDO treatment increases SOCE and Orai1 expression in neonatal rat ventricular cardiomyocytes (Sabourin et al., 2016); we measured Orai1 protein expression by immunoblotting. Our results show that Orai1 protein expression was similar in both experimental conditions (Supplementary Figure 3). Considering that the stromal interaction molecule (STIM)/Orai system is involved in SOCE (Trebak and Putney, 2017), we determined the mRNA levels of both proteins. However, no changes in mRNA levels of *Orai1* and *Stim1* were observed in ALDO-treated arteries (normalized *Orai1* mRNA levels: 1.0 ± 0.16, *n* = 4 control vs. 0.72 ± 0.11, *n* = 4 ALDO-treated samples, *P* = 0.2084; normalized *Stim1* mRNA levels: 1.0 ± 0.08, in *n* = 4 control vs. 1.15 ± 0.16, *n* = 4 ALDO-treated samples, *P* = 0.4217). These results do not rule out the participation of additional mechanisms of Ca^2+^ entry (for instance, Ca_v_3.2 and TRP channels) (Evans, 2017; Trebak and Putney, 2017; Fan et al., 2018) which might be altered by ALDO treatment and deserve further studies. Taken together, these data support the conclusion that Ca_v_1.2 contributes to refilling intracellular Ca^2+^ stores in MASMCs via SERCA pump activity. To rule out the possibility that changes in SR Ca^2+^ load were associated with ALDO-induced modifications in the expression of calsequestrin-2 (CSQ2), a key SR Ca^2+^ buffering protein (Dagnino-Acosta and Guerrero-Hernández, 2009; Esfandiarei et al., 2013), we measured CSQ2 expression in Aldo-treated and untreated MAs. No differences were found in the expression of CSQ2 (Normalized CSQ2/GAPDH expression: 1.00 ± 0.09 in the control group vs. 1.14 ± 0.26 in the ALDO-treated group; *P* = 0.6; *N* = 5 for each experimental condition. Supplementary Figure 4).

### Aldosterone Treatment Increases Ca^2+^ Spark Frequency in Mesenteric Artery Smooth Muscle Cells

**FIGURE 4:**
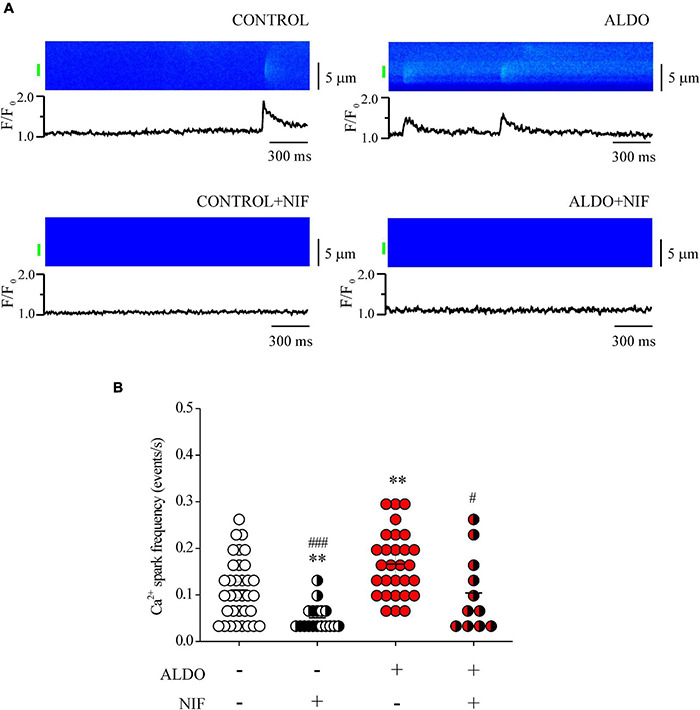
Aldosterone treatment increases Ca^2+^ spark frequency in MASMCs. **(A)** Representative pseudo-colored confocal images of Ca^2+^ sparks (*top*) and normalized (*F*/*F*_0_) fluorescence profiles (*bottom*) from Fluo 4-loaded MASMCs treated or not with aldosterone (ALDO 10 nM, 24 h) and preincubated or not with Nifedipine (1 μM, 10 min). The fluorescence profile was calculated in the region indicated by the green bar. **(B)** Scatterplot with mean ± SEM illustrates Ca^2+^ spark frequency in Fluo 4-loaded MASMCs from control (*empty circles*, *n* = 35 cells/*N* = 5 rats); ALDO-treated arteries (*red circles*, *n* = 29 cells/*N* = 4 rats); pre-incubated with Nifedipine (NIF, *white/black circles, n* = 18 cells/*N* = 4 rats; and *red/black circles, n* = 11 cells/*N* = 3 rats, respectively). ^**^P < 0.01 vs. control cells. ^#^*P* < 0.05 and ^###^*P* < 0.001 vs. ALDO-treated group.

Mesenteric artery smooth muscle cells exhibit Ca^2+^ sparks reflecting localized Ca^2+^ release through type 2 RyR, the predominant isoform of RyRs in these cells (Krishnamoorthy et al., 2014; Matsuki et al., 2018). Given that Ca_v_1.2-mediated Ca^2+^ influx contributes to increase SR Ca^2+^ load in MASMCs, and that Ca^2+^ spark ignition is tightly regulated by SR Ca^2+^ load due to a spatial coupling between LTCCs and RyRs, in which SERCA pump must have a role (Cheranov and Jaggar, 2002; Essin et al., 2007; Takeda et al., 2011; Fan et al., 2018), we measured Ca^2+^ sparks. We previously showed that ALDO modified spatio-temporal properties of Ca^2+^ sparks in cardiomyocytes (Gómez et al., 2009); therefore, we analyzed the frequency and spatial properties of Ca^2+^ sparks in Fluo 4-loaded MASMCs by confocal microscopy. Figure 4A shows representative confocal images and fluorescence profiles of Ca^2+^ sparks recorded in ALDO-treated MASMCs and control cells, with and without Nifedipine (1 μM) to block Ca_v_1.2-mediated Ca^2+^ entry. We have found a significant increase in Ca^2+^ spark frequency in MASMCs treated with ALDO (Figure 4B and Table 2) an effect blocked in presence of Nifedipine (Figure 4B and Table 2). In-depth analysis of Ca^2+^ sparks characteristics revealed a Nifedipine-dependent reduction of basal fluorescence (F_0_) in control MASMCs, an effect that has been previously observed with diltiazem, a benzothiazepine class of LTCC channel blocker (Cheranov and Jaggar, 2002). In ALDO-treated and untreated cells, Nifedipine also reduced the duration of Ca^2+^ sparks (Table 2). The coincubation of MASMCs with ALDO and the selective MR inhibitor RU28318 prevented the increase in Ca^2+^ spark frequency induced by ALDO treatment alone (Ca^2+^ spark frequency in events/s: 0.16 ± 0.1, *n* = 29 ALDO-treated cells/*N* = 4 rats vs. 0.11 ± 0.01, *n* = 30 ALDO + RU28318-treated cells/*N* = 3 rats, *P* < 0.001). These data suggest a direct link between the MR signaling pathway activation, SR Ca^2+^ load and RyR activity in MASMCs. Changes in RyR protein expression or the expression of RyR accessory proteins might alter Ca^2+^ spark frequency and properties in VSMCs (Fernández-Velasco et al., 2014). Moreover, in ALDO-treated cardiac cells the alterations in frequency and properties of Ca^2+^ sparks were associated with downregulation of immunophilin FK506-binding proteins (FKBP12 and 12.6), which regulate RyR activity (Gómez et al., 2009); therefore, we also determined the protein expression levels of three key regulatory proteins of RyR activity in VSMCs: FKBP12.6, sorcin, and calsequestrin (Wang et al., 2004; Rueda et al., 2006; Esfandiarei et al., 2013). Immunoblot results showed a similar expression of RyR2 and its regulatory proteins in both experimental groups (Supplementary Figure 4).

**TABLE 2:** Characteristics of Ca^2+^ spark recorded in untreated (Control) and aldosterone-treated (ALDO) mesenteric artery smooth muscle cells in the absence and presence of Nifedipine (1 μM).

			+ Nifedipine	

	**Control**	**ALDO**	**Control**	**ALDO**
		
**Cells (*n*)**	**35**	**29**	**18**	**11**
Basal fluorescence (*F*_0_)	5.81 ± 0.52	5.90 ± 0.53	3.58 ± 0.25^##^	5.78 ± 0.56
Frequency (events/s)	0.11 ± 0.01	0.17 ± 0.01**	0.05 ± 0.01**,###	0.10 ± 0.02^#^
Amplitude (*F*/*F*_0_)	1.89 ± 0.05	1.77 ± 0.03	2.10 ± 0.09	1.91 ± 0.07
FWHM (μm)	1.88 ± 0.07	1.89 ± 0.06	2.05 ± 0.15	1.87 ± 0.12
FDHM (ms)	45.97 ± 2.36	50.25 ± 2.09	42.62 ± 4.88^#^	37.55 ± 6.10^*,###^
Rising time (ms)	21.80 ± 1.40	23.67 ± 1.14	18.37 ± 1.73	19.12 ± 2.48
Decay time (ms)	84.82 ± 16.83	79.91 ± 9.43	70.63 ± 23.22	35.29 ± 4.53

Values are mean ± SEM of indicated recorded cells (n) for each experimental condition. Confocal images of Ca^2+^ sparks were recorded with a laser scanning confocal microscope in line scan mode (five images per cell of 1000 lines each, at speed of 1.92 ms/line). Basal fluorescence intensity (F_0_) was calculated inside the cell as the average fluorescence intensity of those pixels without sparks. Ca^2+^ spark data were obtained from nCTL = 191 events, nCTL + Nif = 35 events, nALDO = 315 events, and nALDO + Nif = 50 events.*P ≤ 0.05, and **P ≤ 0.01 vs. control condition. ^#^P ≤ 0.05, ^##^P ≤ 0.01, and ^###^P ≤ 0.001 vs. ALDO-treated cells. FDHM, full duration at half maximum, FWHM, full width at half maximum.

### Aldosterone Treatment Increases Spontaneous Transient Outward Current Frequency in Mesenteric Artery Smooth Muscle Cells

**FIGURE 5:**
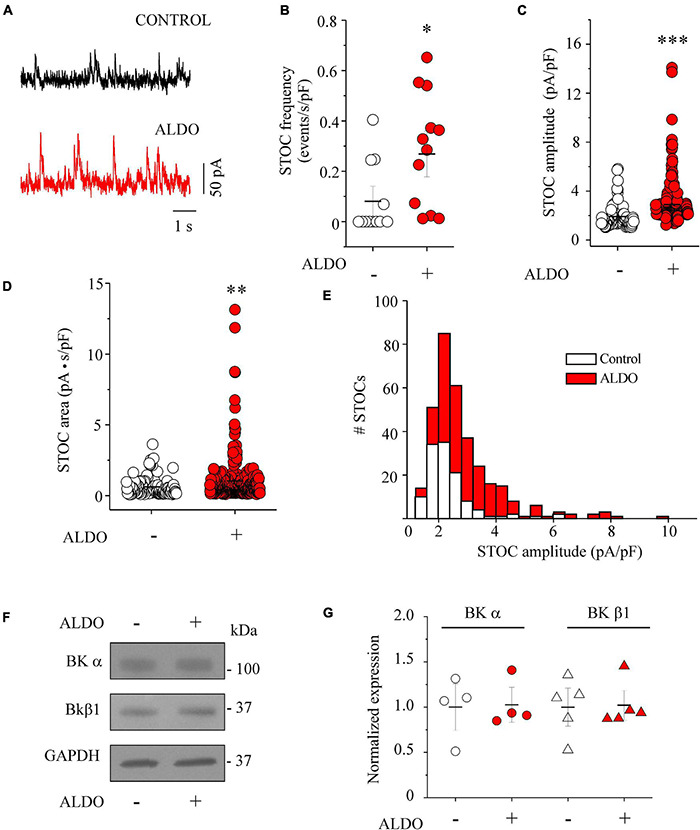
Aldosterone treatment increases the frequency and the amplitude of STOCs in MASMCs without modifying BK channel subunit expression. **(A)** Representative traces of STOCs recorded at a holding potential of –40 mV from MASMCs in the absence (CONTROL, *black trace*) or after 24 h-treatment with aldosterone 10 nM (ALDO, *red trace*). Scatterplots with mean ± SEM illustrate STOC frequency (**B**, normalized with respect to cell capacitance, in events/s/pF), STOC amplitude (**C**, normalized with respect to cell capacitance, in pA/pF), and STOC area-under-the-curve (**D**, in pA.s) in control MASMCs (*n* = 119 events/*n* = 12 cells/*N* = 4 animals, *empty circles*) and ALDO-treated cells (*n* = 333 events/*n* = 12 cells/*N* = 5 animals, *red circles*). *P < 0.05, ^**^P < 0.01, and ^***^P < 0.001 vs. control group. **(E)** Histogram distribution of normalized STOC amplitudes in control (*n* = 119 events/*n* = 12 cells/*N* = 4 rats, *white bars*) and ALDO-treated MASMCs (*n* = 333 events/*n* = 12 cells/*N* = 5 rats, *red bars*) indicates the increase in the amplitude of STOCs above 3.6 pA/pF in ALDO-treated cells. **(F,G)** Representative immunoblot images and scatterplot with mean ± SEM of BK_Ca_ channel α subunit expression (*n* = 4 control samples, *empty circles*; *n* = 4 ALDO-treated samples, *red circles*), and β1 subunit expression (*n* = 5 control samples, *empty triangles*; *n* = 5 ALDO-treated samples, *red triangles*). Each sample was prepared with a pool of 3–4 MA segments from three rats. Values were normalized with respect to GAPDH expression.

In VSMCs, Ca^2+^ sparks exert a negative feedback effect on contractility by decreasing LTCC-mediated Ca^2+^ entry due to the activation of BK_Ca_ channel and STOCs generation (Gollasch et al., 1998). Particularly, in resistance-sized MAs, spark-activated BK_Ca_ channels oppose vasoconstriction (Krishnamoorthy et al., 2014), an effect also observed in cerebral arteries (Knot et al., 1998; Jaggar et al., 2000). In addition, BK_Ca_ channels have been recognized as targets of ALDO-induced MR activation in vascular cells (Liu et al., 1995; Ambroisine et al., 2007). Firstly, to assess the effect of ALDO treatment on cell size, we measured cell capacitance to estimate membrane surface area of MASMCs, as an indirect index of cell dimension. No difference in membrane capacitance was found in MASMCs of both experimental groups (in pF: 13.0 ± 1.1, *n* = 12 control cells vs. 12.38 ± 0.54, *n* = 12 ALDO-treated cells). Thus, we studied whether the increase in Ca^2+^ spark frequency in ALDO-treated MASMCs could have an impact on BK_Ca_ channel activity by analyzing STOC frequency and amplitude at near resting membrane potential (−40 mV) in PSS. Figure 5A shows representative STOC recordings in MASMCs elicited at −40 mV. The STOC frequency was higher in ALDO-treated MASMCs than in control cells (Figure 5B). Moreover, STOC amplitude (Figure 5C) and area-under-the-curve (Figure 5D) were significantly increased in ALDO-treated MASMCs. Figure 5E shows a histogram distribution of STOC amplitudes. Aldosterone promoted the increase of STOC events with amplitudes of >3.6 pA/pF. Considering that membrane depolarization of MASMCs increased the frequency and amplitude of STOCs (Pucovský and Bolton, 2006), we measured the voltage dependence of STOC frequency and amplitude in MASMCs of both experimental groups. In ALDO-treated cells, STOC properties were significantly augmented at depolarizing holding potentials of −40 mV, −20 mV, and 0 mV, with respect to control MASMCs (Supplementary Figure 5). Because STOCs result from BK_Ca_ channel activation (Pérez et al., 1999; Krishnamoorthy et al., 2014), we determined the effect of ALDO treatment on BK channel subunit expression. Figure 5F shows representative immunoblots of both the pore-forming α subunit and the β1 accessory subunit of BK_Ca_ channels. Protein expression of both BK_Ca_ channel subunits was similar between ALDO-treated and control MAs (Figure 5G). Therefore, the augmented STOC activity was not due to changes in BK_Ca_ channel expression and could be attributed to the increase of Ca^2+^ spark frequency and SR Ca^2+^ load in ALDO-treated cells.

### Aldosterone Enhances Acetylcholine-Induced Vasorelaxation of Mesenteric Arteries

**FIGURE 6:**
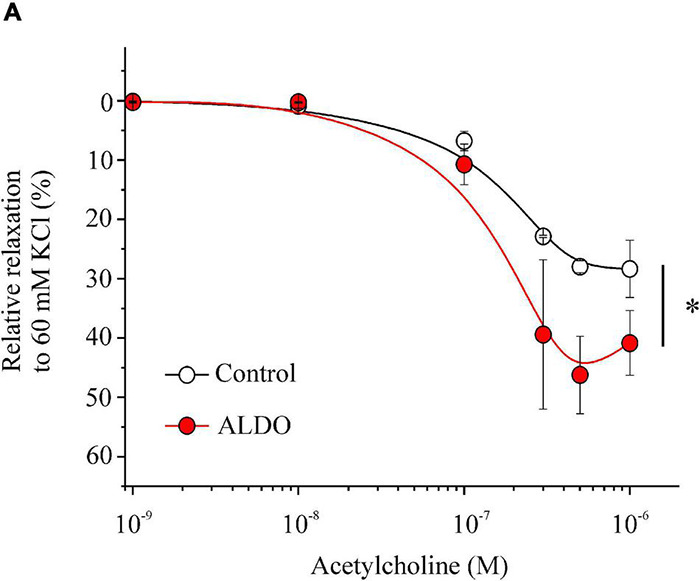
Enhanced acetylcholine-mediated vasorelaxation of ALDO-treated MAs. Relaxation responses of MAs treated (ALDO, *red circles*) or not (CONTROL, *empty circles*) with aldosterone (10 nM, 24 h) to increasing concentrations of acetylcholine (ACh, from 0.001 to 1 μM). MA rings were pre-contracted with KCl 60 mM and exposed to cumulative ACh concentrations (*n* = 6 MA rings/*N* = 6 rats, for each experimental condition). Relaxation is shown as a percentage with respect to the contraction response produced by 60 mM KCl, which was taken as 100%. Data are shown as mean ± SEM. *P < 0.05 vs. non-linear data fit of control arteries.

Large-conductance Ca^2+^-activated K^+^ channels through STOCs play an important role in the regulation of vascular tone by hyperpolarizing MASMCs and inducing vasorelaxation (Krishnamoorthy et al., 2014). The functional coupling between Ca^2+^ sparks and STOCs promotes vasorelaxation by decreasing LTCC-mediated Ca^2+^ entry. Thus, we determine whether vasorelaxation was enhanced in our experimental model. As shown in Figure 6, in 60 mM KCl-precontracted MA rings the vasorelaxation response to ACh was augmented in ALDO-treated MA rings compared to control arteries (EC_50_ for ACh-induced relaxation in mM: 0.69 ± 0.10, *n* = 6 control arteries vs. 0.37 ± 0.04, *n* = 6 ALDO-treated arteries; *P* ≤ 0.05). However, the maximal relaxation response to ACh showed a tendency to be higher in ALDO-treated MA rings with respect to control arteries; though, this tendency was not significant as illustrated by the *P* value (Maximal relaxation in %: 28.3 ± 4.8, *n* = 6 control arteries vs. 40.8 ± 5.4, *n* = 6 ALDO-treated arteries, *P* = 0.1162); These data suggest that ALDO treatment increased the sensitivity to ACh, but not the maximal relaxation response to this agonist.

## Discussion

**FIGURE 7:**
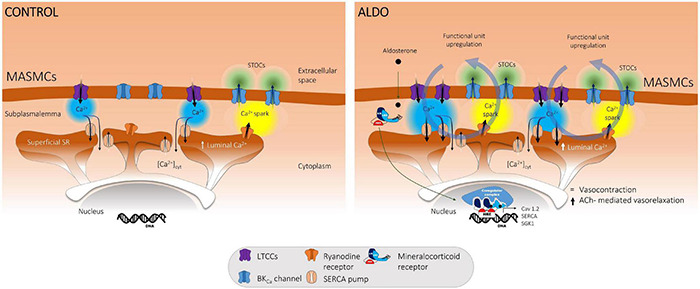
Proposed model for ALDO-mediated upregulation of the functional unit that controls Ca^2+^ dynamics at the SR-PM nanodomain of MASMCs. In normal conditions (CONTROL, *left*) K^+^-mediated membrane depolarization induces a Ca^2+^ influx via Ca_v_1.2 (LTCCs). This Ca^2+^ entry is buffered by the SERCA pump toward the luminal SR Ca^2+^ reservoirs activating RyRs (Ca^2+^ sparks) and BK_Ca_ channels (STOCs). Ca_v_1.2, SERCA pump, RyRs and BK_Ca_ channels work as a functional unit in the PM-SR nanodomain regulating [Ca^2+^]_cyt_, luminal SR Ca^2+^ levels, and opposing vasoconstriction. The treatment of MAs with aldosterone (ALDO, *right*) increases Ca_v_1.2 protein expression and induces higher Ca^2+^ entry in MASMCs. However, the depolarization-induced vascular contraction was not enhanced because of the upregulation of this functional unit, which involves increased expression and activity of SERCA pump controlling abnormal Ca^2+^ influx at the PM-SR nanodomain, increasing SR Ca^2+^ content, Ca^2+^ spark and STOC frequencies, opposing to depolarization-induced vasoconstriction and enhancing ACh-mediated vasorelaxation.

In this study, we describe the crucial role of the SERCA pump in buffering Ca_v_1.2-mediated Ca^2+^ entry in ALDO-treated MASMCs. Our work shows that a short-term (24 h) *ex vivo* exposure to 10 nM ALDO of resistance MAs: (1) increased both Ca_v_1.2 and SERCA pump protein expressions; (2) increased depolarization-induced Ca^2+^ influx, luminal SR Ca^2+^ load, and Ca^2+^ spark and STOC frequencies; in consequence, (3) depolarization-induced vasoconstriction was unaffected as well global [Ca^2+^]_cyt_; and (4) ACh-mediated vasorelaxation was enhanced. In the presence of TGN, the reintroduction of extracellular Ca^2+^ induced a significant increase in cytoplasmic Ca^2+^ levels, unmasking the key participation of the SERCA pump in counterbalancing Ca^2+^ influx and avoiding undesired increments of global [Ca^2+^]_cyt_. All these data allow us to propose the model depicted in Figure 7. Under physiological conditions (Control, *left side*), Ca_v_1.2-mediated Ca^2+^ influx is directed by SERCA pump to the SR Ca^2+^ stores; this, in turn, promotes the ignition of Ca^2+^ sparks (*via* clusters of RyRs) and STOCs (through BK_Ca_ channel activation). Therefore, Ca_v_1.2, SERCA pump, RyRs and BK_Ca_ channels are actively working as a functional unit at the PM-SR nanodomain, regulating [Ca^2+^]_cyt_, luminal SR Ca^2+^ levels, and opposing to vasoconstriction. The exposure to 10 nM aldosterone (ALDO, *right side*) increases Ca_v_1.2 protein expression and induces higher Ca_v_1.2-mediated Ca^2+^ influx in MASMCs. However, the depolarization-induced vascular contraction is not enhanced because of SERCA pump upregulation, which efficiently counterbalances Ca^2+^ entry at the PM-SR nanodomain, increasing SR Ca^2+^ content, Ca^2+^ spark and STOC frequencies, and enhancing ACh-mediated vasorelaxation. The net result of this new steady-state is higher Ca^2+^ cycling at the PM-SR nanodomain, dampening unsought elevations of [Ca^2+^]_cyt_. However, higher luminal SR Ca^2+^ levels might also participate in enhancing receptor-mediated Ca^2+^ release and promote abnormal vasoconstriction, a hallmark feature of overactive ALDO/MR signaling pathway in chronic pathological conditions.

Cumulative evidence supports the physiological role of MR signaling in MAs. Highly specific binding of [^3^H]-aldosterone has been observed in rat mesenteric vascular arcade (Funder et al., 1989). In addition, MR, aldosterone synthase and 11-BHSD2 activity, key proteins of the local ALDO system are found in MAs (Takeda et al., 1993, 1997). Because over-activation of the MR/ALDO signaling pathway has been associated with vascular dysfunction (Schiffrin, 2006); it is thus reasonable to consider that either the chronic exposure to pathological levels of ALDO or the increased expression of vascular MR might promote vascular damage, endothelial dysfunction, and altered vasorelaxation (Virdis et al., 2002; Pu et al., 2003; Schiffrin, 2006; Nguyen Dinh Cat et al., 2010). More recent evidence from our laboratories has shown that the activation of the ALDO/RM pathway upregulates Ca_v_1.2 expression in MAs (Mesquita et al., 2018); however, the effect of ALDO-induced MR activation on key Ca^2+^ handling proteins of the PM-SR nanodomain, such as SERCA pump, RyRs, and BK_Ca_ channels has remained elusive; and this work demonstrates that the compensatory increased expression of SERCA pump counterbalanced the higher activity of Ca_v_1.2 channels.

Increased vascular resistance and vascular reactivity could be attributable to the thickening of the vascular walls as part of the maladaptive vessel changes in hypertension (Folkow, 1978). Therefore, to rule out the effects of chronic ALDO-mediated maladaptive vessel changes; and to avoid unsought Angiotensin II-induced vascular MR activation (Jaffe and Mendelsohn, 2005); we studied the effects of ALDO in rat MAs *ex vivo* following a previously described protocol (Mesquita et al., 2018) and our results demonstrate that short-term treatment with ALDO induced changes involving SERCA pump upregulation which precedes all the chronic maladaptive effects in MAs.

The precise control of [Ca^2+^]_cyt_ in MASMCs is crucial for regulating their physiological activity; and LTCCs have a prominent role in Ca^2+^ entry, regulating myogenic tone, arterial diameter, and BP (Moosmang et al., 2003; Fan et al., 2018). In spontaneous hypertensive rats (SHR), the chronic elevation of ALDO induces the upregulation of LTCCs in MAs, which has been associated with increased Ca^2+^ influx and vascular reactivity (Cox and Lozinskaya, 1995; Matsuda et al., 1997; Pratt et al., 2002). However, contrary to what might have been expected, we found no increase in depolarization-induced vasoconstriction, as previously reported in coronary arteries exposed to ALDO under similar experimental conditions (Mesquita et al., 2018). One explanation for this discrepancy could be distinct molecular targets activated by the ALDO/MR signaling pathway between vascular tissues. For instance, SERCA pump expression is augmented in mesenteric arteries but, to our knowledge, there is no data available about SERCA pump expression in coronary arteries exposed to ALDO in similar conditions used in this work (10 nM, 24 h). Therefore, we propose that SERCA efficiently counterbalances abnormal Ca^2+^ influx in some types of arteries (i.e., mesenteric arteries) but probably not in others (i.e., coronary arteries). This idea is further supported by reports showing that when LTCCs are activated through K^+^-mediated membrane depolarization maneuvers, vasoconstriction responses of mesenteric and cerebral arteries are unaffected even in conditions of ALDO/MR signaling pathway over-activation (Suzuki et al., 1994; Xavier et al., 2008; Chrissobolis et al., 2014).

Once membrane depolarization is initiated, several processes act in concert to control [Ca^2+^]_cyt_ elevation, including (1) negative feedback mechanisms that decrease Ca^2+^ influx; for instance, Ca^2+^-dependent inactivation of LTTCs, and BK_Ca_ channel activation which induces membrane hyperpolarization and decreases the open probability of LTCCs; (2) cytosolic Ca^2+^ buffering by proteins such as sorcin and calmodulin; and (3) Ca^2+^ removal mechanisms at the PM-SR nanodomain which include the SERCA pump, the Na^+^/Ca^2+^ exchanger, and the plasma membrane Ca^2+^ ATPase (Kamishima and McCarron, 1996; Van Breemen et al., 2013; Evans, 2017). Our data support the notion that SERCA pump, RyR2, and BK_Ca_ channels act simultaneously to control abnormal Ca_v_1.2-mediated Ca^2+^ influx in MAs treated with ALDO. Importantly, our data also highlight the crucial role of the superficial buffer barrier in SMCs in controlling Ca^2+^ influx. Van Breemen et al. (1995) have postulated the existence of a superficial and fenestrated surface of the SR separated from the plasma membrane by a narrow space that generates a barrier to Ca^2+^ entry *via* Ca_v_1.2 channels. Then, extracellular Ca^2+^ is effectively captured toward the luminal SR Ca^2+^ stores by the SERCA pump limiting its access to the bulk myoplasm (Chen and van Breemen, 1993; Van Breemen et al., 1995; Van Breemen et al., 2013). Because SMC contraction relies on the increment of [Ca^2+^]_cyt_ due to Ca^2+^ influx and Ca^2+^ release from SR Ca^2+^ stores (Flores-Soto et al., 2013); and occurs only when actin-myosin myofilaments are activated by Ca^2+^ reaching the deep myoplasm, not by Ca^2+^ localized in the space between the plasma membrane and the superficial SR (Van Breemen et al., 1995), it is possible to reduce the force of contraction when the rate of Ca^2+^ influx is controlled (Casteels and Droogmans, 1981). Interestingly, vasoconstriction response to α-adrenoceptor is augmented in MAs of DOCA-salt rats but not depolarization-induced contraction (Perry and Webb, 1991; Suzuki et al., 1994) supporting the idea that contraction is related more to the rate than to the extent of Ca^2+^ entry (Casteels and Droogmans, 1981).

L-type voltage-dependent Ca^2+^ channels also contribute to refilling luminal SR Ca^2+^ load via SERCA pump activity and are involved in the formation of Ca^2+^ sparks, which induce vasorelaxation (Cheranov and Jaggar, 2002; Krishnamoorthy et al., 2014; Fan et al., 2018). In fact, the tight coupling between Ca_v_1.2 and RyR is not required for Ca_v_1.2 to initiate Ca^2+^ sparks in MASMCs. Instead, the Ca_v_1.2 channel contributes to [Ca^2+^]_cyt_, which in turn activates the SERCA pump, increases SR Ca^2+^ load, and triggers Ca^2+^ sparks (Essin et al., 2007; Fan et al., 2018). Interestingly, the Ca_v_3.2 channel is also involved in the ignition of Ca^2+^ sparks, though by a direct mechanism in which its localization in caveolae and close apposition to RyRs is crucial to trigger Ca^2+^ sparks in MASMCs. Despite Ca_v_3.2 channel has a smaller participation in the generation of Ca^2+^ sparks with respect to Ca_v_1.2 channel (Fan et al., 2018); additional work is required to delineate its specific contribution to Ca^2+^ spark ignition in ALDO-treated MASMCs.

Thus, in our experimental model, we hypothesize that Ca_v_1.2 is the predominant pathway to provide Ca^2+^ for loading SR Ca^2+^ stores; and that the SERCA pump has a crucial role in the control of Ca_v_1.2-mediated Ca^2+^ influx. In agreement with this idea, we demonstrate that blocking Ca^2+^ entry with Nifedipine significantly reduced Ca^2+^ spark frequency, suggesting that the SERCA pump mediates Ca^2+^ store refilling and, in consequence, Ca^2+^ spark generation.

Reports about ALDO effects on the expression and activity of the SERCA pump are scarce. In this regard, a study has shown that the treatment of human aortic SMCs with ALDO decreased SERCA2a transcription (Chou et al., 2015). Specifically, this work demonstrated that 48-h ALDO exposure (10 nM and 100 nM) reduced SERCA2 protein expression and SERCA2a mRNA levels. Aldosterone inhibited the expression of SERCA2a through MR-dependent mitochondrial DNA-specific transcription factors TFAM and TFB2M (Chou et al., 2015). Our work also provides evidence for an MR-mediated genomic pathway inducing the increase of SERCA pump mRNA levels. However, we did not examine the transcription factor involved in this response. An alternative mechanism could be that increased intracellular Ca^2+^ levels promote SERCA pump expression in VSMCs (Wu et al., 2001). Previous works have shown that resting [Ca^2+^]_cyt_ is augmented (1.6-fold over controls) in aortic SMCs of ALDO-salt hypertensive rats (Liu et al., 1995), which might increase SERCA pump expression (Levitsky et al., 1993). However, our results demonstrate that [Ca^2+^]_cyt_ is not elevated in ALDO-treated MASMCs, arguing against this mechanism. Moreover, an MR antagonist blocked the ALDO-mediated increase in SERCA pump mRNA and protein expression; therefore, an MR-mediated, tissue-specific genomic pathway is involved. Given that rat MAs express SERCA2a and SERCA2b isoforms (Lagaud et al., 1999), our data do not clarify whether one or both isoforms responded to ALDO treatment. We think that SERCA2a might be sensitive to ALDO because this isoform increases the rate of Ca^2+^ store refilling in VSMCs, maintaining a high SR Ca^2+^ concentration (Bobe et al., 2011). However, this hypothesis awaits further studies.

Regarding ALDO effects on RyR expression and activity, this work is the first to report that short-term treatment with ALDO increases Ca^2+^ spark frequency in MASMCs. Previously, we have demonstrated that ALDO/MR signaling pathway activation augmented Ca^2+^ spark frequency and altered Ca^2+^ spark properties in cardiomyocytes, which was associated with the downregulation of FKBP12 and FKBP12.6, accessory proteins of the RyR macromolecular complex (Gómez et al., 2009). Likely, we found an increase in Ca^2+^ spark frequency of ALDO-treated MASMCs but FKBP12.6 expression remained unchanged; therefore, this does not explain the change in Ca^2+^ spark frequency. In our hands, the increase in Ca^2+^ spark frequency was associated with the increased SR Ca^2+^ load in ALDO-treated MAs, because the addition of Nifedipine, an LTCC blocker, effectively reduced the frequency of these local Ca^2+^ events, even in ALDO-treated cells. This result agrees with previous publications (Cheranov and Jaggar, 2002; Fan et al., 2018). Diltiazem belongs to the benzothiazepine subclass of Ca^2+^ channel antagonists and blocks Ca_v_1.2 in resting state, but membrane depolarization enhances its inhibitory effect (Tang et al., 2019). This attribute explains the inhibitory effect on Ca^2+^ sparks in VSMC under resting, non-depolarizing conditions (bath solution containing 6 mM K^+^) (Cheranov and Jaggar, 2002). Nifedipine belongs to the dihydropyridine subclass of LTCC blockers and exhibits prominent a voltage-dependent antagonism, according to which its potency increases with the level of depolarization. This justifies, in part, the general vascular selectivity of Nifedipine over other Ca^2+^ channel blockers (Triggle, 1991). Consistent with this feature, in the present work, Nifedipine reduced the frequency and duration of Ca^2+^ sparks in both experimental groups under mild-depolarized conditions (PSS-20K).

Depolarization of MASMCs increased the frequency and amplitude of STOCs and elicited Ca^2+^ sparks from Ca^2+^ discharge regions (Pucovský and Bolton, 2006). Moreover, in VSMCs of the transgenic mouse with VSMC-specific Ca_v_1.2 channel gene inactivation (SMAKO mouse), both frequency and amplitude of STOCs were reduced, together with decreased cytosolic Ca^2+^ levels and SR Ca^2+^ load (Essin et al., 2007). Accordingly, we observed an increase in STOC and spark activities associated with higher Ca_v_1.2 and SERCA pump protein expressions. Although, Ca^2+^ influx through Ca_v_1.2 channels is the primary source of Ca^2+^ for triggering Ca^2+^ sparks (70–80%) in MASMCs; an additional Ca^2+^ source is provided by Ca_v_3.2 channels (20–30%) (Fan et al., 2018). Besides, higher micromolar concentrations of Nifedipine may interfere with T-type Ca^2+^ channel activity (Abd El-Rahman et al., 2013). Therefore, future work is needed to delineate the specific contribution of Ca_v_1.2 and Ca_v_3.2 to Ca^2+^ spark generation in ALDO-treated MASMCs.

Existing evidence shows an association between ALDO/MR signaling pathway, vascular BK_Ca_ channel expression, and activity. In a mouse model with cardiomyocyte-specific overexpression of the aldosterone synthase gene (MAS mice), an impairment in ACh-induced vasorelaxation, associated with decreased mRNA expression of α and β1 subunits of BK_Ca_ channels in coronary arteries has been reported (Ambroisine et al., 2007). Although BK_Ca_ channel protein expression levels were not determined, the β1 subunit of the BK_Ca_ channel showed a similar expression in immunostainings of freshly isolated coronary arteries (Ambroisine et al., 2007). In contrast, in the SMC-MR-KO mouse no difference was found in mRNA expression of α and β1 subunits of BK_Ca_ channel in aorta (McCurley et al., 2012); similarly to our results in coronary arteries treated with ALDO (Mesquita et al., 2018). Further experiments with the SMC-MR-KO mouse will be helpful to corroborate our results.

In contrast with previous results in coronary arteries (Mesquita et al., 2018), we found a higher sensitivity of ACh-mediated vasorelaxation after 24-h ALDO treatment. This result can be explained by the increased SERCA-mediated SR Ca^2+^ load, and augmented Spark-STOC activity. On the other hand, chronic effects of ALDO can lead to an impairment of endothelium-dependent vasorelaxation of MAs (Xavier et al., 2008). The pre-treatment of chronically exposed MAs to ALDO with indomethacin (a cyclooxygenase inhibitor), resulted in a recovery of the sensitivity to ACh without modification of the ACh-mediated maximal vasodilation response (Xavier et al., 2008). The latter similar to our results, suggesting the lack of endothelial damage in our experimental conditions. Rapid vasodilator action of ALDO has been described to depend on MR receptor activation, enhancing the production of nitric oxide (NO) (Davel et al., 2017). Therefore, endothelial MRs (EC-MR) could potentially contribute to ALDO effects in MAs. However, the EC-MR may not be involved in the enhanced ACh-induced vasorelaxation observed in our experimental model of ALDO-treated MAs, based on the following considerations. First, EC-MR-mediated vasodilator actions of ALDO occur in minutes after the mineralocorticoid application (Davel et al., 2017), while our study evaluates ALDO effects after 24-h treatment. Second, we have shown previously that the endothelial layer is not involved in ALDO effects on coronary and aorta vasoconstriction (Mesquita et al., 2018). Third, it has been suggested that EC-MR does not play a significant role in the control of vascular function in non-disease states (Mueller et al., 2015). Finally, NO-mediated relaxation of MAs from a mouse model over-expressing EC-MR was similar to control (Nguyen Dinh Cat et al., 2010). Interestingly, EC protection is lost when cardiovascular risk factors are present (Jaffe and Jaisser, 2014; Davel et al., 2017; DuPont and Jaffe, 2017). Therefore, the role of EC-MR in MAs deserves more studies, specifically under pathological conditions.

This work highlights the intricacies of tissue-specific MR signaling and the differential actors of MR signaling across vascular beds, contributing to ALDO-associated impairments of vascular functions and blood flow control. Initial functional changes induced by ALDO are adaptative, but in chronic pathological conditions will become maladaptive, leading to poor vascular function, vascular remodeling, and poor compliant arteries as observed in hypertension; thus, further delineation of the MR-induced molecular pathways that control vascular SERCA2 expression will require additional analysis.

### New and Noteworthy

Cumulative evidence has shown that the mineralocorticoid receptor (MR) is found in vascular tissues where regulates expression and activity of ion channels that participate in Ca^2+^ handling of smooth muscle cells (SMCs), but none of these studies had evaluated the effect of ALDO/MR signaling pathway on the functional unit that regulates vascular function, comprising Ca_v_1.2, SERCA pump, Ryanodine receptors, and BK_Ca_ channels, and we aimed to study it comprehensively. Our work provides novel evidence about ALDO-induced upregulation of this functional unit unveiling the crucial role of the SERCA pump in counterbalancing Ca_v_1.2-mediated Ca^2+^ influx at the subplasmalemmal space of mesenteric artery SMCs. This work highlights the intricacies of tissue-specific MR signaling and the differential actors of the ALDO/MR signaling pathway across vascular beds, contributing to ALDO-associated impairments of vascular functions. Initial functional changes induced by ALDO are adaptative, but in chronic pathological conditions, they would become maladaptive leading to vascular dysfunction, vascular remodeling, and poor compliant arteries as observed in hypertension.

## Data Availability Statement

The raw data supporting the conclusions of this article will be made available by the authors, without undue reservation.

## Ethics Statement

The animal study was reviewed and approved by COMITÉ INTERNO PARA EL CUIDADO Y USO DE LOS ANIMALES DE LABORATORIO (CICUAL) Cinvestav.

## Author Contributions

RS-E contributed to the conceptualization, methodology, data collecting, data analysis, and writing. AG-H contributed to the conceptualization, resources, review and editing. AG contributed to the conceptualization, resources, funding acquisition, and review and editing. J-PB contributed to the conceptualization, methodology, resources, funding acquisition, and review and editing. AR contributed to the conceptualization, methodology, data analysis, writing, resources, funding acquisition, and review and editing. All authors contributed to the article and approved the submitted version.

## Conflict of Interest

The authors declare that the research was conducted in the absence of any commercial or financial relationships that could be construed as a potential conflict of interest.

## Publisher’s Note

All claims expressed in this article are solely those of the authors and do not necessarily represent those of their affiliated organizations, or those of the publisher, the editors and the reviewers. Any product that may be evaluated in this article, or claim that may be made by its manufacturer, is not guaranteed or endorsed by the publisher.

## Abbreviations

ALDO: aldosterone
MA: mesenteric arteries
MASMC: mesenteric artery smooth muscle cell
LTCC: L-type voltage-dependent Ca^2+^ channels
SMC: smooth muscle cell
ACh: acetylcholine
ALDO: aldosterone
BK_Ca_ channel: large-conductance Ca^2+^-activated K^+^ channel
CASQ2: calsequestrin-2
DMEM: Dulbecco’s Modified Eagle Medium
FKBP12.6: 12.6 kDa FK506-binding protein
GAPDH: glyceraldehyde-3-phosphate dehydrogenase
LTCC: voltage-gated L-type Ca_v_1.2 channel
MA: mesenteric artery
MASMC: mesenteric artery smooth muscle cell
MR: mineralocorticoid receptor
Nif: Nifedipine
*Orai1*: calcium release-activated calcium channel protein 1
PM-SR: plasma membrane-sarcoplasmic reticulum
PSS: physiological saline solution
RyR: ryanodine receptor
*Rpl32*: 60S ribosomal protein L32
s*m22*: smooth muscle protein 22-alpha
SERCA: Sarco/Endoplasmic reticulum Ca^2+^ pump
*Sgk1*: serum/glucocorticoid regulated kinase 1
SMC: smooth muscle cell
*Stim1*: stromal interaction molecule 1
SOCE: store-operated calcium entry
STOC: spontaneous transient outward current
TGN: thapsigargin
VSMC: vascular smooth muscle cell
*Ywhaz*: 14-3-3 protein zeta/delta
11-BHSD2: 11-beta-hydroxysteroid dehydrogenase type 2.

## References

[B1] Abd El-Rahman R. R. Harraz O. F. Brett S. E. Anfinogenova Y. Mufti R. E. Goldman D. (2013). Identification of L- and T-type Ca2+ channels in rat cerebral arteries: role in myogenic tone development. *Am. J. Physiol. Heart Circ. Physiol.* 304 H58–H71. 10.1152/AJPHEART.00476.2012 23103495 PMC3543679

[B2] Acelajado M. C. Hughes Z. H. Oparil S. Calhoun D. A. (2019). Treatment of resistant and refractory hypertension. *Circ. Res.* 124 1061–1070. 10.1161/CIRCRESAHA.118.31S2156 30920924 PMC6469348

[B3] Ambroisine M. L. Favre J. Oliviero P. Rodriguez C. Gao J. Thuillez C. (2007). Aldosterone-induced coronary dysfunction in transgenic mice involves the calcium-activated potassium (BKCa) channels of vascular smooth muscle cells. *Circulation* 116 2435–2443. 10.1161/CIRCULATIONAHA.107.722009 17984374

[B4] Bartoli F. Bailey M. A. Rode B. Mateo P. Antigny F. Bedouet K. (2020). Orai1 channel inhibition preserves left ventricular systolic function and normal Ca2+ handling after pressure overload. *Circulation* 141 199–216. 10.1161/CIRCULATIONAHA.118.038891 31906693 PMC6970549

[B5] Bénitah J. P. Vassort G. (1999). Aldosterone upregulates Ca2+ current in adult rat cardiomyocytes. *Circ. Res.* 85 1139–1145. 10.1161/01.RES.85.12.1139 10590240

[B6] Bobe R. Hadri L. Lopez J. J. Sassi Y. Atassi F. Karakikes I. (2011). SERCA2a controls the mode of agonist-induced intracellular Ca2+ signal, transcription factor NFAT and proliferation in human vascular smooth muscle cells. *J. Mol. Cell. Cardiol.* 50 621–633. 10.1016/J.YJMCC.2010.12.016 21195084 PMC3062203

[B7] Briet M. Barhoumi T. Mian M. O. R. Coelho S. C. Ouerd S. Rautureau Y. (2016). Aldosterone-induced vascular remodeling and endothelial dysfunction require functional angiotensin type 1a receptors. *Hypertension* 67 897–905. 10.1161/HYPERTENSIONAHA.115.07074 27045029 PMC4833572

[B8] Casteels R. Droogmans G. (1981). Exchange characteristics of the noradrenaline-sensitive calcium store in vascular smooth muscle cells or rabbit ear artery. *J. Physiol.* 317 263–279. 10.1113/jphysiol.1981.sp013824 7310734 PMC1246788

[B9] Chen Q. van Breemen C. (1993). The superficial buffer barrier in venous smooth muscle: sarcoplasmic reticulum refilling and unloading. *Br. J. Pharmacol.* 109 336–343. 10.1111/j.1476-5381.1993.tb13575.x 8358539 PMC2175673

[B10] Cheranov S. Y. Jaggar J. H. (2002). Sarcoplasmic reticulum calcium load regulates rat arterial smooth muscle calcium sparks and transient K Ca currents. *J. Physiol.* 544 71–84. 10.1113/jphysiol.2002.025197 12356881 PMC2290569

[B11] Chou C. H. Chen Y. H. Hung C. S. Chang Y. Y. Tzeng Y. L. Wu X. M. (2015). Aldosterone impairs vascular smooth muscle function: from clinical to bench research. *J. Clin. Endocrinol. Metab.* 100 4339–4347. 10.1210/jc.2015-2752 26401591

[B12] Chrissobolis S. Drummond G. R. Faraci F. M. Sobey C. G. (2014). Chronic aldosterone administration causes Nox2-mediated increases in reactive oxygen species production and endothelial dysfunction in the cerebral circulation. *J. Hypertens.* 32 1815–1821. 10.1097/HJH.0000000000000259 24991871 PMC4151299

[B13] Cox R. H. Lozinskaya I. M. (1995). Augmented calcium currents in mesenteric artery branches of the spontaneously hypertensive rat. *Hypertension* 26 1060–1064. 10.1161/01.HYP.26.6.1060 7498968

[B14] Dagnino-Acosta A. Guerrero-Hernández A. (2009). Variable luminal sarcoplasmic reticulum Ca(2^+^) buffer capacity in smooth muscle cells. *Cell Calcium* 46 188–196. 10.1016/j.ceca.2009.07.005 19679350

[B15] Davel A. P. Anwar I. J. Jaffe I. Z. (2017). The endothelial mineralocorticoid receptor: mediator of the switch from vascular health to disease. *Curr. Opin. Nephrol. Hypertens.* 26 97–104. 10.1097/MNH.0000000000000306 27930384 PMC5382958

[B16] de Alba-Aguayo D. R. Pavón N. Mercado-Morales M. Miranda-Saturnino M. López-Casamichana M. Guerrero-Hernández A. (2017). Increased calcium leak associated with reduced calsequestrin expression in hyperthyroid cardiomyocytes. *Cell Calcium* 62 29–40. 10.1016/j.ceca.2017.01.009 28169003

[B17] DuPont J. J. Jaffe I. Z. (2017). The role of the mineralocorticoid receptor in the vasculature. *J. Endocrinol.* 234 T67–T82. 10.1530/JOE-17-0009 28634267 PMC5518626

[B18] Esfandiarei M. Fameli N. Choi Y. Y. H. Tehrani A. Y. Hoskins J. G. van Breemen C. (2013). Waves of calcium depletion in the sarcoplasmic reticulum of vascular smooth muscle cells: an inside view of spatiotemporal Ca2+ regulation. *PLoS One* 8:e55333. 10.1371/journal.pone.0055333 23408969 PMC3567057

[B19] Essin K. Gollasch M. (2009). Role of ryanodine receptor subtypes in initiation and formation of calcium sparks in arterial smooth muscle: comparison with striated muscle. *J. Biomed. Biotechnol.* 2009:135249. 10.1155/2009/135249 20029633 PMC2793424

[B20] Essin K. Welling A. Hofmann F. Luft F. C. Gollasch M. Moosmang S. (2007). Indirect coupling between Ca v 1.2 channels and ryanodine receptors to generate Ca2+ sparks in murine arterial smooth muscle cells. *J. Physiol.* 584 205–219. 10.1113/jphysiol.2007.138982 17673505 PMC2277062

[B21] Evans A. M. (2017). *Nanojunctions of the Sarcoplasmic Reticulum Deliver Site- and Function-Specific Calcium Signaling in Vascular Smooth Muscles*, 1st Edn. Amsterdam: Elsevier Inc, 10.1016/bs.apha.2016.10.001 28212795

[B22] Fan G. Kaßmann M. Hashad A. M. Welsh D. G. Gollasch M. (2018). Differential targeting and signalling of voltage-gated T-type Cav3.2 and L-type Cav1.2 channels to ryanodine receptors in mesenteric arteries. *J. Physiol.* 596 4863–4877. 10.1113/JP276923 30146760 PMC6187032

[B23] Fernández-Velasco M. Ruiz-Hurtado G. Gómez A. M. Rueda A. (2014). Ca2+ handling alterations and vascular dysfunction in diabetes. *Cell Calcium* 56 397–407. 10.1016/j.ceca.2014.08.007 25218935

[B24] Flores-Soto E. Reyes-García J. Sommer B. Montaño L. M. (2013). Sarcoplasmic reticulum Ca2+ refilling is determined by L-type Ca2+ and store operated Ca2+ channels in guinea pig airway smooth muscle. *Eur. J. Pharmacol.* 721 21–28. 10.1016/j.ejphar.2013.09.060 24113526

[B25] Folkow B. (1978). Cardiovascular structural adaptation; its role in the initiation and maintenance of primary hypertension. *Clin. Sci.* 55 3s–22s. 10.1042/cs055003s 153216

[B26] Funder J. W. Pearce P. T. Smith R. Campbell J. (1989). Vascular type I aldosterone binding sites are physiological mineralocorticoid receptors. *Endocrinology* 125 2224–2226. 10.1210/endo-125-4-2224 2551643

[B27] Ganitkevich V. Isenberg G. (1990). Isolated guinea pig coronary smooth muscle cells. *Circ. Res.* 67 525–529. 10.1161/01.res.67.2.525 2376084

[B28] Ghosh D. Syed A. U. Prada M. P. Nystoriak M. A. Santana L. F. Nieves-Cintrón M. (2017). Calcium channels in vascular smooth muscle. *Adv. Pharmacol.* 78 49–87. 10.1016/bs.apha.2016.08.002 28212803 PMC5439506

[B29] Gollasch M. Wellman G. C. Knot H. J. Jaggar J. H. Damon D. H. Bonev A. D. (1998). Ontogeny of local sarcoplasmic reticulum Ca2+ signals in cerebral arteries: Ca2+ sparks as elementary physiological events. *Circ. Res.* 83 1104–1114. 10.1161/01.res.83.11.1104 9831705

[B30] Gómez A. M. Rueda A. Sainte-Marie Y. Pereira L. Zissimopoulos S. Zhu X. (2009). Mineralocorticoid modulation of cardiac ryanodine receptor activity is associated with downregulation of FK506-binding proteins. *Circulation* 119 2179–2187. 10.1161/CIRCULATIONAHA.108.805804 19364981

[B31] Gomez-Sanchez C. E. De Rodriguez A. F. Romero D. G. Estess J. Warden M. P. Gomez-Sanchez M. T. (2006). Development of a panel of monoclonal antibodies against the mineralocorticoid receptor. *Endocrinology* 147 1343–1348. 10.1210/EN.2005-0860 16293659

[B32] Grynkiewicz G. Poenie M. Tsien R. Y. (1985). A new generation of Ca2+ indicators with greatly improved fluorescence properties. *J. Biol. Chem.* 260 3440–3450. 3838314

[B33] Jaffe I. Z. Jaisser F. (2014). Endothelial cell mineralocorticoid receptors: turning cardiovascular risk factors into cardiovascular dysfunction. *Hypertens* 63 915–917. 10.1161/HYPERTENSIONAHA.114.01997 24566083 PMC3984296

[B34] Jaffe I. Z. Mendelsohn M. E. (2005). Angiotensin II and aldosterone regulate gene transcription via functional mineralocortocoid receptors in human coronary artery smooth muscle cells. *Circ. Res.* 96 643–650. 10.1161/01.RES.0000159937.05502.d1 15718497

[B35] Jaggar J. H. Porter V. A. Jonathan Lederer W. Nelson M. T. (2000). Calcium sparks in smooth muscle. *Am. J. Physiol. Cell Physiol.* 278 C235–C256. 10.1152/ajpcell.2000.278.2.c235 10666018

[B36] Jaggar J. H. Wellman G. C. Heppner T. J. Porter V. A. Perez G. J. Gollasch M. (1998). Ca2+ channels, ryanodine receptors and Ca(2+)-activated K+ channels: a functional unit for regulating arterial tone. *Acta Physiol. Scand.* 164 577–587. 10.1046/j.1365-201X.1998.00462.x 9887980

[B37] Kamishima T. McCarron J. G. (1996). Depolarization-evoked increases in cytosolic calcium concentration in isolated smooth muscle cells of rat portal vein. *J. Physiol.* 492(Pt 1), 61–74. 10.1113/jphysiol.1996.sp021289 8730583 PMC1158861

[B38] Kim P. J. Cole M. A. Kalman B. A. Spencer R. L. (1998). Evaluation of RU28318 and RU40555 as selective mineralocorticoid receptor and glucocorticoid receptor antagonists, respectively: receptor measures and functional studies. *J. Steroid Biochem. Mol. Biol.* 67 213–222. 10.1016/s0960-0760(98)00095-8 9879980

[B39] Knot H. J. Standen N. B. Nelson M. T. (1998). Ryanodine receptors regulate arterial diameter and wall [Ca2+] in cerebral arteries of rat via Ca2+-dependent K+ channels. *J. Physiol.* 508 211–221. 10.1111/j.1469-7793.1998.211br.x 9490841 PMC2230867

[B40] Krishnamoorthy G. Sonkusare S. K. Heppner T. J. Nelson M. T. (2014). Opposing roles of smooth muscle BK channels and ryanodine receptors in the regulation of nerve-evoked constriction of mesenteric resistance arteries. *Am. J. Physiol. Hear. Circ. Physiol.* 306:H981. 10.1152/ajpheart.00866.2013 24508642 PMC3962638

[B41] Lagaud G. J. Randriamboavonjy V. Roul G. Stoclet J. C. Andriantsitohaina R. (1999). Mechanism of Ca2+ release and entry during contraction elicited by norepinephrine in rat resistance arteries. *Am. J. Physiol.* 276 H300–H308. 9887044 10.1152/ajpheart.1999.276.1.H300

[B42] Lalevée N. Rebsamen M. C. Barrère-Lemaire S. Perrier E. Nargeot J. Bénitah J. P. (2005). Aldosterone increases T-type calcium channel expression and *in vitro* beating frequency in neonatal rat cardiomyocytes. *Cardiovasc. Res.* 67 216–224. 10.1016/j.cardiores.2005.05.009 15919070

[B43] Leung F. P. Yung L. M. Yao X. Laher I. Huang Y. (2008). Store-operated calcium entry in vascular smooth muscle. *Br. J. Pharmacol.* 153 846–857. 10.1038/sj.bjp.0707455 17876304 PMC2267267

[B44] Levitsky D. Clergue M. Lambert F. Souponitskayas V. Jemtelfl T. H. Le Lecarpentier Y. (1993). Sarcoplasmic reticulum calcium transport and Ca2+ -ATPase gene expression in thoracic and abdominal aortas of normotensive and spontaneously hypertensive rats. *J. Biol. Chem.* 268 8325–8331. 10.1016/s0021-9258(18)53099-4 7681842

[B45] Liu Y. Jones A. W. Sturek M. (1995). Ca(2+)-dependent K+ current in arterial smooth muscle cells from aldosterone-salt hypertensive rats. *Am. J. Physiol.* 269 H1246–H1257. 7485555 10.1152/ajpheart.1995.269.4.H1246

[B46] Lombès M. Oblin M. E. Gasc J. M. Baulieu E. E. Farman N. Bonvalet J. P. (1992). Immunohistochemical and biochemical evidence for a cardiovascular mineralocorticoid receptor. *Circ. Res.* 71 503–510. 10.1161/01.res.71.3.503 1323429

[B47] Mangelsdorf D. J. Thummel C. Beato M. Herrlich P. Schütz G. Umesono K. (1995). The nuclear receptor superfamily: the second decade. *Cell* 83 835–839. 10.1016/0092-8674(95)90199-x 8521507 PMC6159888

[B48] Matsuda K. Lozinskaya I. Cox R. H. (1997). Augmented contributions of voltage-gated Ca2+ channels to contractile responses in spontaneous hypertensive rat mesenteric arteries. *Am. J. Hypertens.* 7061 1231–1239. 10.1016/s0895-7061(97)00225-2 9397241

[B49] Matsuki K. Kato D. Takemoto M. Suzuki Y. Yamamura H. Ohya S. (2018). Negative regulation of cellular ca2+ mobilization by ryanodine receptor type 3 in mouse mesenteric artery smooth muscle. *Am. J. Physiol. Cell Physiol.* 315 C1–C9. 10.1152/ajpcell.00006.2018 29537866

[B50] McCurley A. Pires P. W. Bender S. B. Aronovitz M. Zhao M. J. Metzger D. (2012). Direct regulation of blood pressure by smooth muscle cell mineralocorticoid receptors. *Nat. Med.* 18 1429–1433. 10.1038/nm.2891 22922412 PMC3491085

[B51] Mesquita T. R. Auguste G. Falcón D. Ruiz-Hurtado G. Salazar-Enciso R. Sabourin J. (2018). Specific activation of the alternative cardiac promoter of cacna1c by the mineralocorticoid receptor. *Circ. Res.* 122 e49–e61. 10.1161/CIRCRESAHA.117.312451 29467196

[B52] Moosmang S. Schulla V. Welling A. Feil R. Feil S. Wegener J. W. (2003). Dominant role of smooth muscle L-type calcium channel Cav1.2 for blood pressure regulation. *EMBO J.* 22 6027–6034. 10.1093/emboj/cdg583 14609949 PMC275441

[B53] Mueller K. B. Bender S. B. Hong K. Yang Y. Aronovitz M. Jaisser F. (2015). Endothelial mineralocorticoid receptors differentially contribute to coronary and mesenteric vascular function without modulating blood pressure. *Hypertension* 66 988–997. 10.1161/HYPERTENSIONAHA.115.06172 26351033 PMC4600033

[B54] Nelson M. T. Worley J. F. (1989). Dihydropyridine inhibition of single calcium channels and contraction in rabbit mesenteric artery depends on voltage. *J. Physiol.* 412 65–91. 10.1113/jphysiol.1989.sp017604 2481035 PMC1190564

[B55] Nelson M. T. Cheng H. Rubart M. Santana L. F. Bonev A. D. Knot H. J. (1995). Relaxation of arterial smooth muscle by calcium sparks. *Science* 270 633–637. 10.1126/science.270.5236.633 7570021

[B56] Nguyen Dinh Cat A. Griol-Charhbili V. Loufrani L. Labat C. Benjamin L. Farman N. (2010). The endothelial mineralocorticoid receptor regulates vasoconstrictor tone and blood pressure. *FASEB J.* 24 2454–2463. 10.1096/fj.09-147926 20299606

[B57] Pérez G. J. Bonev A. D. Patlak J. B. Nelson M. T. (1999). Functional coupling of ryanodine receptors to KCa channels in smooth muscle cells from rat cerebral arteries. *J. Gen. Physiol.* 113 229–238. 10.1085/jgp.113.2.229 9925821 PMC2223357

[B58] Perry P. A. Webb R. C. (1991). Agonist-sensitive calcium stores in arteries from steroid hypertensive rats. *Hypertension* 17 603–611. 10.1161/01.HYP.17.5.603 2022405

[B59] Pfaffl M. W. (2001). A new mathematical model for relative quantification in real-time RT-PCR. *Nucleic Acids Res.* 29:e45. 10.1093/nar/29.9.e45 11328886 PMC55695

[B60] Pratt P. F. Bonnet S. Ludwig L. M. Bonnet P. Rusch N. J. (2002). Upregulation of L-type Ca2+ channels in mesenteric and skeletal arteries of SHR. *Hypertension* 40 214–219. 10.1161/01.HYP.0000025877.23309.36 12154116

[B61] Pu Q. Neves M. F. Virdis A. Touyz R. M. Schiffrin E. L. (2003). Endothelin antagonism on aldosterone-induced oxidative stress and vascular remodeling. *Hypertension* 42 49–55. 10.1161/01.HYP.0000078357.92682.EC 12782645

[B62] Pucovský V. Bolton T. B. (2006). Localisation, function and composition of primary Ca(2+) spark discharge region in isolated smooth muscle cells from guinea-pig mesenteric arteries. *Cell Calcium* 39 113–129. 10.1016/J.CECA.2005.10.002 16297446

[B63] Romero-García T. Landa-Galvan H. V. Pavón N. Mercado-Morales M. Valdivia H. H. Rueda A. (2020). Autonomous activation of CaMKII exacerbates diastolic calcium leak during beta-adrenergic stimulation in cardiomyocytes of metabolic syndrome rats. *Cell Calcium* 91:102267. 10.1016/j.ceca.2020.102267 32920522 PMC7530131

[B64] Rueda A. Fernández-Velasco M. Benitah J.-P. Gómez A. M. (2013). Abnormal Ca2+ spark/STOC coupling in cerebral artery smooth muscle cells of obese type 2 diabetic mice. *PLoS One* 8:e53321. 10.1371/journal.pone.0053321 23301060 PMC3536748

[B65] Rueda A. García L. Guerrero-Hernández A. (2002). Luminal Ca2+ and the activity of sarcoplasmic reticulum Ca2+ pumps modulate histamine-induced all-or-none Ca2+ release in smooth muscle cells. *Cell. Signal.* 14 517–527. 10.1016/s0898-6568(01)00284-4 11897492

[B66] Rueda A. Song M. Toro L. Stefani E. Valdivia H. H. (2006). Sorcin modulation of Ca^2+^ sparks in rat vascular smooth muscle cells. *J. Physiol.* 576 887–901. 10.1113/jphysiol.2006.113951 16931553 PMC1890400

[B67] Sabourin J. Bartoli F. Antigny F. Gomez A. M. Benitah J.-P. P. (2016). Transient Receptor Potential Canonical (TRPC)/Orai1-dependent Store-operated Ca2+ Channels: new targets of aldosterone in cardiomyocytes. *J. Biol. Chem.* 291 13394–13409. 10.1074/jbc.M115.693911 27129253 PMC4933247

[B68] Salazar-Enciso R. Camacho-Concha N. A. Mesquita T. R. Falcón D. Benitah J.-P. Gomez A.-M. (2018). “Mineralocorticoid receptor in calcium handling of vascular smooth muscle cells,” in *Calcium and Signal Transduction*, eds Buchholz J. (London: InTech), 1–21.

[B69] Schiffrin E. L. (1992). Reactivity of small blood vessels in hypertension: relation with structural changes. *Hypertension* 19 II1–II9. 10.1161/01.HYP.19.2_Suppl.II1-a 1735561

[B70] Schiffrin E. L. (2006). Effects of Aldosterone on the Vasculature. *Hypertension* 47 312–318. 10.1161/01.HYP.0000201443.63240.a7 16432039

[B71] Suzuki S. Takata Y. Kubota S. Ozaki S. Kato H. (1994). Characterization of the alpha-1 adrenoceptors in the mesenteric vasculature from deoxycorticosterone-salt hypertensive rats: studies on vasoconstriction, radioligand binding and postreceptor events. *J. Pharmacol. Exp. Ther.* 268 576–583. 8113968

[B72] Takeda Y. Miyamori I. Inaba S. Furukawa K. Hatakeyama H. Yoneda T. (1997). Vascular aldosterone in genetically hypertensive rats. *Hypertension* 29 45–48. 9039078 10.1161/01.hyp.29.1.45

[B73] Takeda Y. Nystoriak M. A. Nieves-Cintrón M. Santana L. F. Navedo M. F. (2011). Relationship between Ca2+ sparklets and sarcoplasmic reticulum Ca2+ load and release in rat cerebral arterial smooth muscle. *Am. J. Physiol. Hear. Circ. Physiol.* 301 H2285–H2294. 10.1152/ajpheart.00488.2011 21984539 PMC3233819

[B74] Takeda Y. Yoneda T. Miyamori I. Gathiram P. Takeda R. (1993). 11β−hidroxysteroid dehydrogenase activity in mesenteric arteries of spontaneously hypertensive rats. *Clin. Exp. Pharmacol. Physiol.* 20 627–631. 10.1111/j.1440-1681.1993.tb01644.x 8261657

[B75] Tang L. Gamal El-Din T. M. Lenaeus M. J. Zheng N. Catterall W. A. (2019). Structural basis for diltiazem block of a voltage-gated Ca 2+ channel. *Mol. Pharmacol.* 96 485–492. 10.1124/MOL.119.117531 31391290 PMC6738535

[B76] Trebak M. Putney J. W. (2017). ORAI calcium channels. *Physiology* 32 332–342. 10.1152/physiol.00011.2017 28615316 PMC5545608

[B77] Triggle D. J. (1991). Sites, mechanisms of action, and differentiation of calcium channel antagonists. *Am. J. Hypertens.* 4 422S–429S. 10.1093/ajh/4.7.422S 1654937

[B78] Van Breemen C. Chen Q. Laher I. (1995). Superficial buffer barrier function of smooth muscle sarcoplasmic reticulum. *Trends Pharmacol. Sci.* 16 98–105. 10.1016/S0165-6147(00)88990-7 7792935

[B79] Van Breemen C. Fameli N. Evans A. M. (2013). Pan-junctional sarcoplasmic reticulum in vascular smooth muscle: nanospace Ca2+ transport for site- and function-specific Ca2+ signalling. *J. Physiol.* 591 2043–2054. 10.1113/jphysiol.2012.246348 23339179 PMC3634518

[B80] Virdis A. Neves M. F. Amiri F. Viel E. Touyz R. M. Schiffrin E. L. (2002). Spironolactone improves angiotensin-induced vascular changes and oxidative stress. *Hypertension* 40 504–510. 10.1161/01.HYP.0000034738.79310.06 12364354

[B81] Wang K. Li H. Xu Y. Shao Q. Yi J. Wang R. (2019). MFEprimer-3.0: quality control for PCR primers. *Nucleic Acids Res.* 47 W610–W613. 10.1093/nar/gkz351 31066442 PMC6602485

[B82] Wang Y.-X. Zheng Y.-M. Mei Q.-B. Wang Q.-S. Collier M. L. Fleischer S. (2004). FKBP12.6 and cADPR regulation of Ca2+ release in smooth muscle cells. *Am. J. Physiol. Cell Physiol.* 286 C538–C546. 10.1152/ajpcell.00106.2003 14592808

[B83] Weinberger M. H. Roniker B. Krause S. L. Weiss R. J. (2002). Eplerenone, a selective aldosterone blocker, in mild-to-moderate hypertension. *Am. J. Hypertens.* 15 709–716. 10.1016/S0895-7061(02)02957-6 12160194

[B84] Wu K. D. Bungard D. Lytton J. (2001). Regulation of SERCA Ca2+ pump expression by cytoplasmic [Ca2+] in vascular smooth muscle cells. *Am. J. Physiol. Cell Physiol.* 280 843–851. 10.1152/ajpcell.2001.280.4.c843 11245601

[B85] Xavier F. E. Aras-López R. Arroyo-Villa I. Del Campo L. Salaices M. Rossoni L. V. (2008). Aldosterone induces endothelial dysfunction in resistance arteries from normotensive and hypertensive rats by increasing thromboxane A 2 and prostacyclin. *Br. J. Pharmacol.* 154 1225–1235. 10.1038/bjp.2008.200 18500359 PMC2483383

