# Supplementary material for: Aldosterone-Induced Sarco/Endoplasmic Reticulum Ca2+ Pump Upregulation Counterbalances Cav1.2-Mediated Ca2+ Influx in Mesenteric Arteries
Source: Front Physiol. 2022 Mar 11;13:834220. doi: 10.3389/fphys.2022.834220 (PMC8963271; doi:10.3389/fphys.2022.834220)
Supplement: Supplementary file 1 [file Data_Sheet_1.docx]

**SUPPLEMENTARY MATERIAL**

**Supplementary Figures**

**
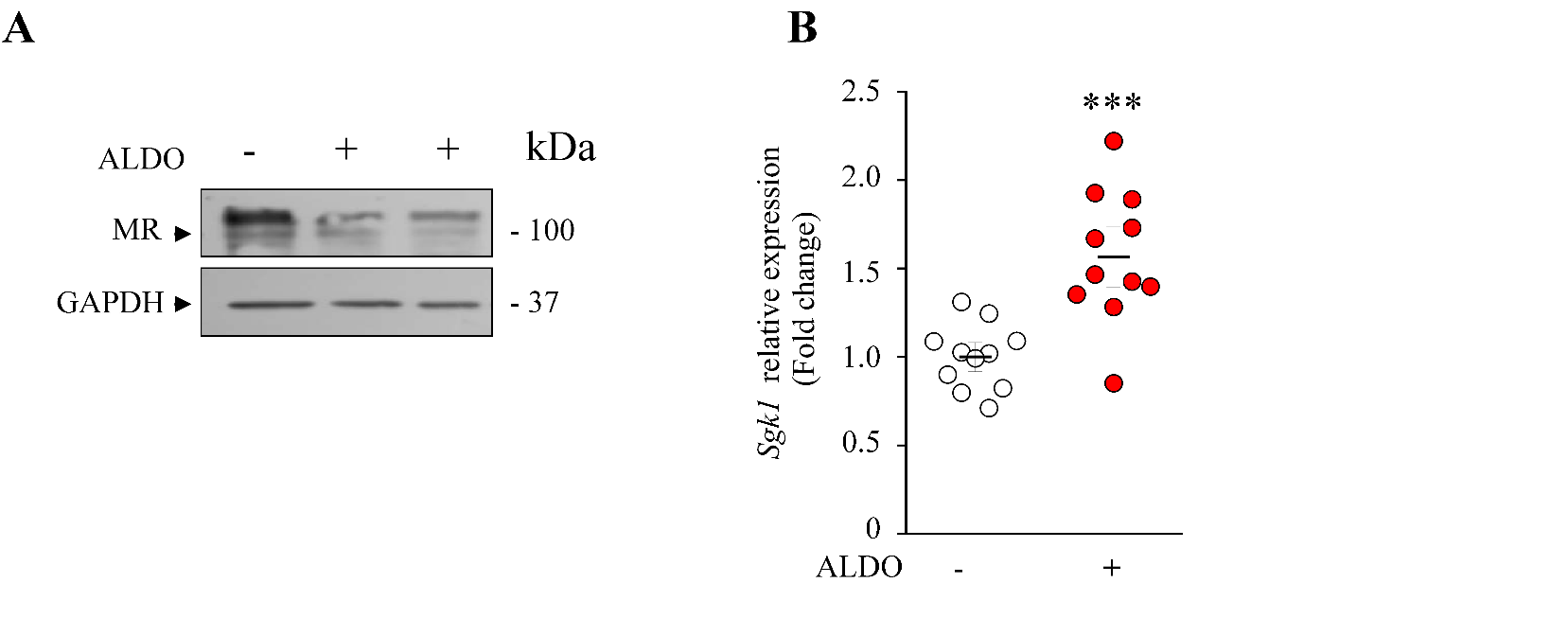
**

**Supplementary Figure 1. Mesenteric arteries have a functional mineralocorticoid receptor**. (**A**) Representative immunoblot images of mineralocorticoid receptor (MR) from mesenteric arteries (MAs) treated with and without aldosterone (10 nM for 24 h, ALDO). The MR has an apparent molecular weight of ~107 kDa in rat MAs. The GAPDH enzyme was used as the loading control. (**B**) ALDO treatment significantly increased the mRNA level of the Serum/glucocorticoid-regulated kinase 1 (*Sgk1*) from MAs (*red circles*), indicating a transcriptionally active MR. Scatterplot also show the mean ± SEM. The Student *t*-test was employed for statistical analysis. *** *P* < 0.001 compared with the control condition (*empty circles*).


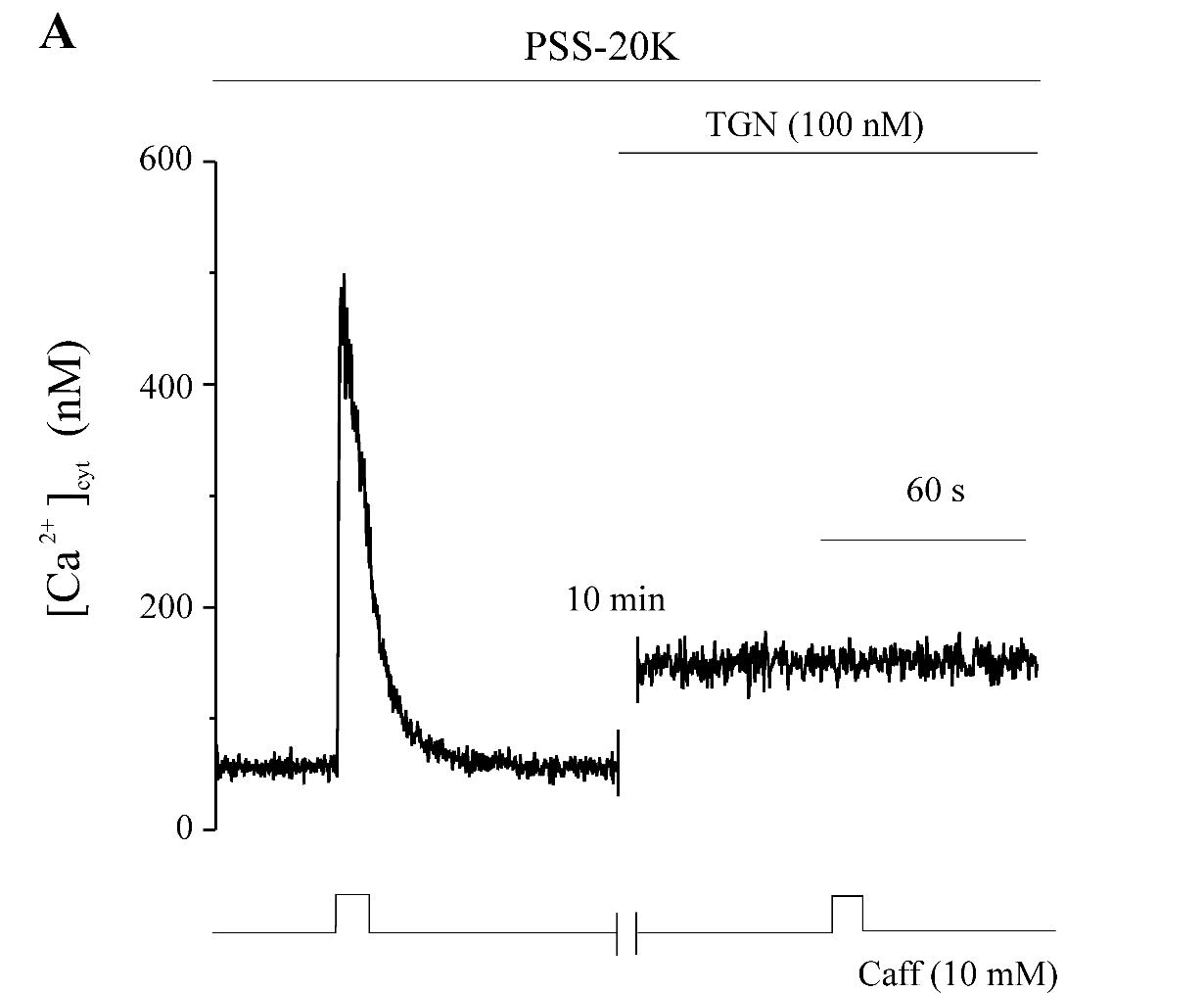


**Supplementary Figure 2. Caffeine induces a thapsigargin-sensitive transient [Ca^2+^]_cyt_ elevation in MASMCs. (A)** Representative recording of [Ca^2+^]_cyt_ from Fura 2-loaded MASMCs bathed in PSS-20K with and without thapsigargin (TGN, 100 nM) as indicated. A puffer pipette was used to apply caffeine (Caff, 10 mM) for the time indicated in the bottom trace. In the absence of TGN (a SERCA pump inhibitor), caffeine produced a transient [Ca^2+^]_cyt_ response. The presence of TGN increased basal [Ca^2+^]_cyt_, and the second application of caffeine (at the indicated time) did not have any effect. These data imply that caffeine-induced transient rise in [Ca^2+^]_cyt_ exclusively represents Ca^2+^ release from the SR Ca^2+^ stores. Also, the application of caffeine solution with the puffer pipette was not activating additional mechanisms of Ca^2+^ entry.


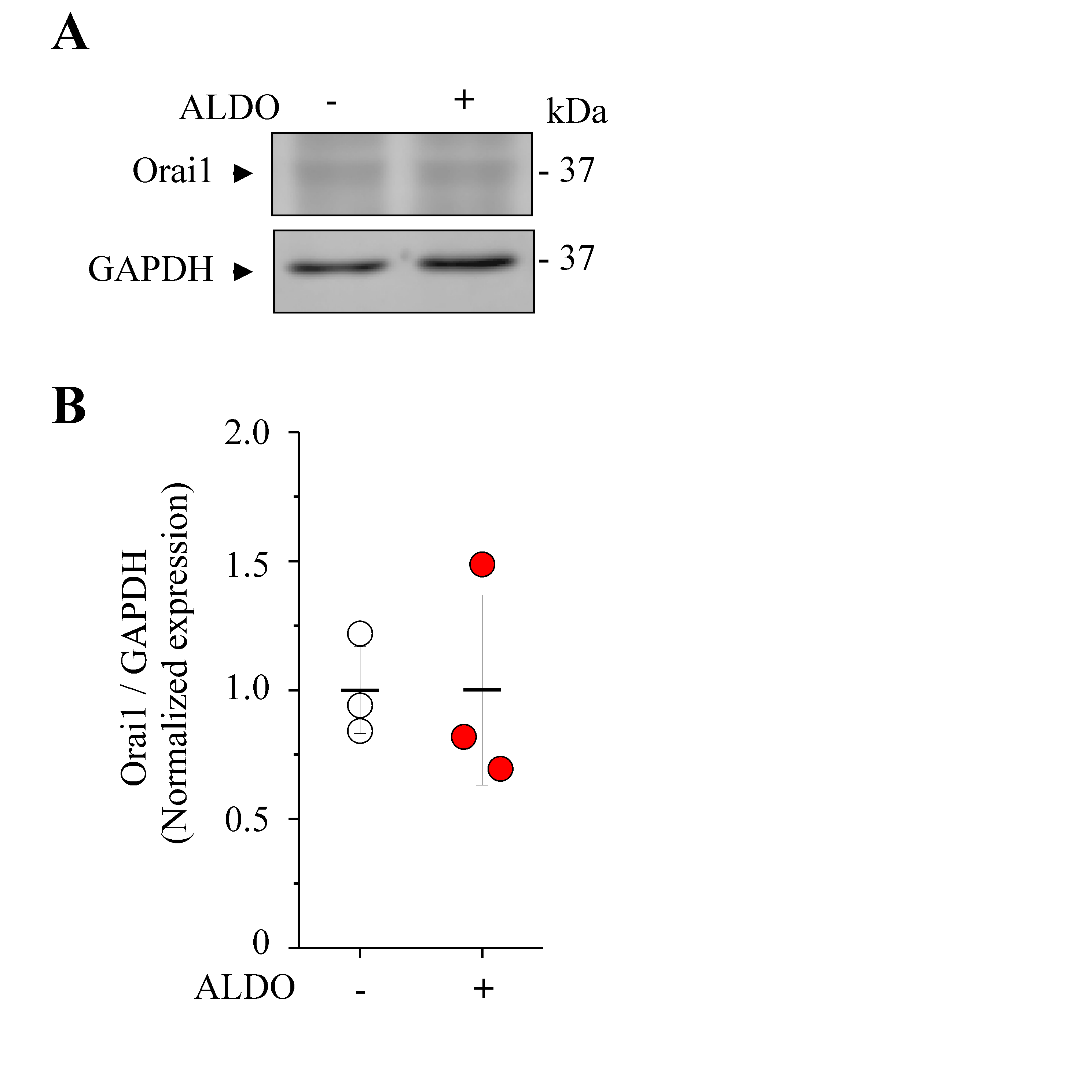


**Supplementary Figure 3**. **Aldosterone treatment does not alter Orai1 protein levels in mesenteric arteries.** (**A**) Representative immunoblot images of Orai1 and GAPDH (as the loading control) from MAs treated with (+) and without (-) aldosterone (10 nM for 24 h). (**B**) Normalized Orai1 expression level with its corresponding GAPDH reference from ALDO-treated MAs (*red circles*) and controls (*empty circles*). The scatterplot also includes the mean ± SEM. Statistical analysis was performed by Student *t* test.


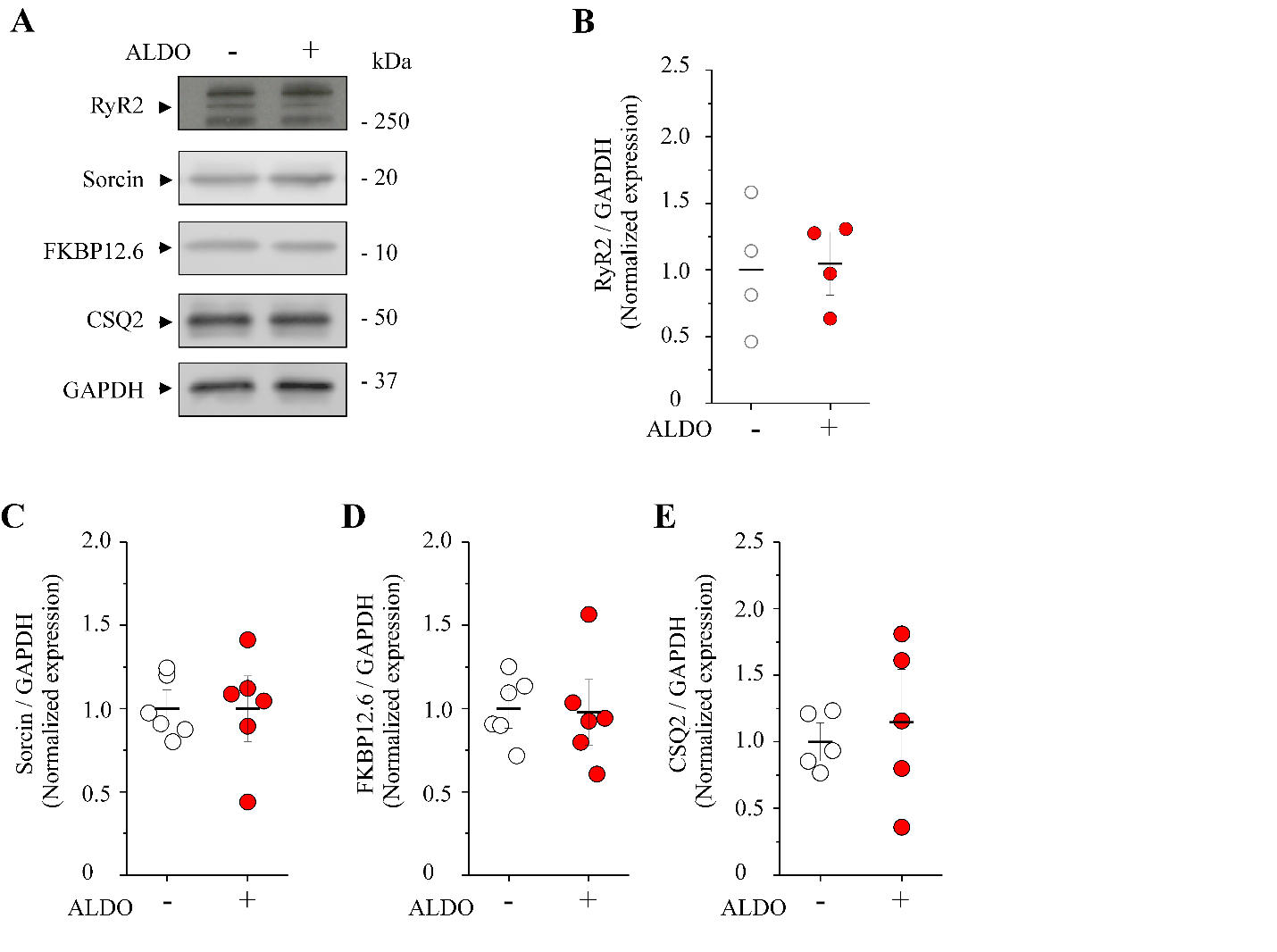


**Supplementary Figure 4. Aldosterone treatment neither changes the expression of RyRs nor its accessory proteins.** (**A**) Representative immunoblot images of Ryanodine receptor type 2 (RyR2) and three of its accessory proteins (sorcin, FKBP12.6, and calsequestrin-2 (CSQ2)) from MAs with (+) and without (-) ALDO (10 nM for 24 h). The GAPDH enzyme was used as the loading control. (**B-E**) Scatterplots also show the mean ± SEM for the normalized expression levels (respect to GAPDH) of RyR2, sorcin, FKBP12.6, and CSQ2, respectively, from MAs treated (*red circles*) or not (*empty circles*) with ALDO. The Student *t-*test was used to determine a significant difference.


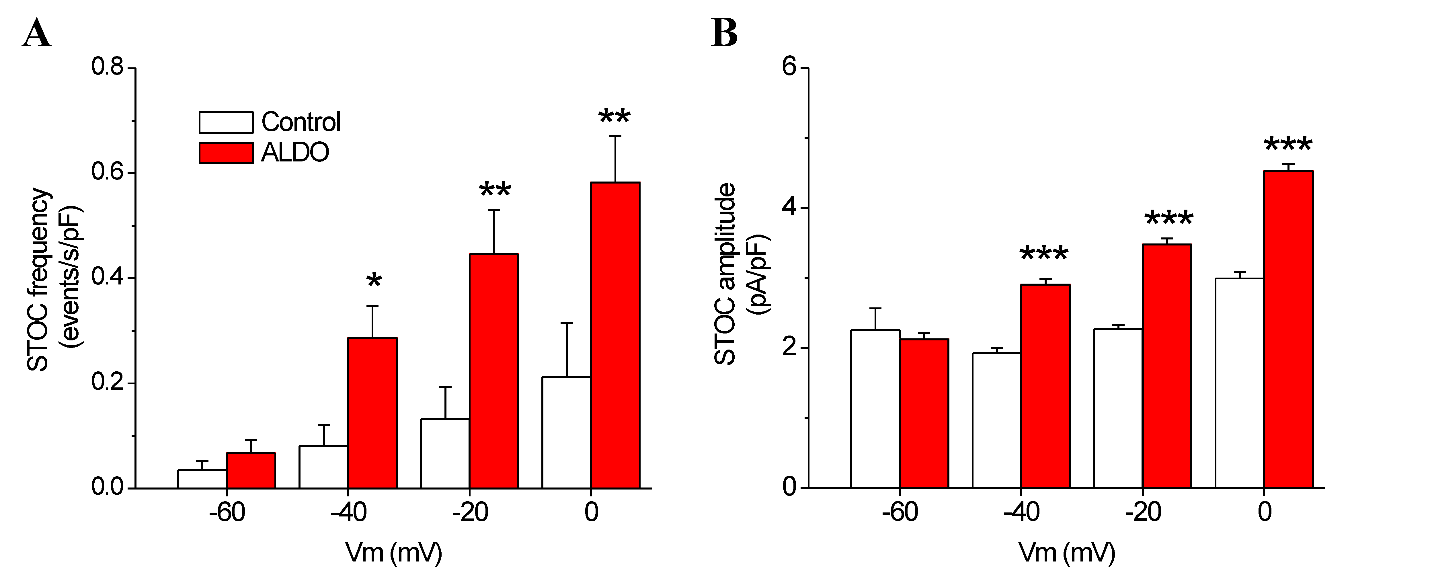


**Supplementary Figure 5. Voltage dependence of frequency and amplitude of STOCs.** Bar graphs of STOC frequency (**A**, in events/s/pF) and STOC amplitude (**B**, in pA/pF) at different holding membrane potentials (Vm) recorded from MASMCs treated with ALDO (n = 12 cells/N = 5 rats, *red bars*) or in its absence (n = 12 cells/N = 4 rats, *white bars*). **P* < 0.05, ***P* < 0.01 and ****P* < 0.001 *vs.* control cells. Statistical analysis was performed by Student *t* test or Mann-Whitney Rank Sum Test when Normality test failed.
